# Neuropeptide Y1 receptor expressing circuit from the central amygdala to lateral hypothalamus modulates binge‐like ethanol consumption in a sex‐dependent manner

**DOI:** 10.1111/acer.70151

**Published:** 2025-08-29

**Authors:** Sophie C. Bendrath, Ashlyn Stone, Anne M. Dankert, Todd E. Thiele

**Affiliations:** ^1^ Department of Psychology and Neuroscience University of North Carolina at Chapel Hill Chapel Hill North Carolina USA; ^2^ Bowles Center for Alcohol Studies University of North Carolina at Chapel Hill Chapel Hill North Carolina USA

**Keywords:** DREADDs, drinking in the dark, neuropeptide Y, NPY overexpression, sex differences

## Abstract

**Background:**

Alcohol use disorder is characterized by maladaptive patterns of alcohol consumption, with emerging evidence suggesting that neuropeptide Y (NPY) signaling through Y1 and Y2 receptors (Y1R and Y2R) within the central amygdala (CeA) plays a critical role in modulating ethanol intake. The current experiments investigate the neural mechanisms underlying binge‐like ethanol drinking, focusing on the involvement of Y1R+ CeA‐to‐lateral hypothalamus (LH) projections, dynamic interactions between Y1R and Y2R within the CeA, and the impact of chronic ethanol exposure on Y1R protein expression.

**Methods:**

NPY1R‐ires‐cre mice received LH cannulation, were infused with cre‐dependent inhibitory (Gi) Designer Receptor Exclusively Activated by Designer Drug (DREADD) or control virus into the CeA, and went through drinking in the dark (DID). Other animals were treated intra‐CeA with an NPY overexpression vector (FIB‐NPY) or control, and went through DID, intermittent access to ethanol (IAE), and open‐field testing. Viral placements and receptor targets were assessed via qPCR. Finally, mice went through six cycles of DID, and Y1R immunohistochemical (IHC) labeling on neurons was assessed for animals sacrificed after the final DID session or after a 24‐h period of abstinence.

**Results:**

Chemogenetic inhibition of Y1R+ CeA‐LH projections selectively reduced binge‐like ethanol drinking in male mice without affecting female mice. Viral NPY overexpression revealed behavioral effects and predictive relationships between receptor mRNA expression and intake patterns. Although no significant differences were found in Y1R/NeuN colocalization across sex and treatment groups, correlational analyses revealed that Y1R expression varied with individual ethanol consumption.

**Conclusions:**

Collectively, these results support a model wherein Y1R signaling within the CeA regulates ethanol consumption through circuit‐specific mechanisms and broader neuroadaptive changes influenced by sex and individual drinking patterns. This research advances our understanding of the neurobiological mechanisms underlying binge‐like ethanol intake and highlights the complex, sex‐dependent roles of NPY‐Y1R and Y2R signaling in the CeA.

## Graphical abstract

Alcohol use disorder involves disrupted neuropeptide Y (NPY) signaling in the central amygdala (CeA). Using chemogenetics, viral NPY overexpression, and immunohistochemistry in male and female mice, we examined the roles of Y1R+ CeA projections to the lateral hypothalamus and CeA Y1R/Y2R dynamics in binge‐like ethanol drinking. Inhibition of CeA‐LH Y1R+ neurons reduced drinking in males, and Y1R/Y2R‐related expression patterns predicted ethanol intake. These findings suggest circuit‐ and sex‐specific mechanisms by which CeA NPY signaling regulates excessive alcohol consumption.

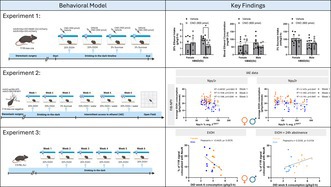

## INTRODUCTION

In the United States, alcohol (ethanol) binge drinking has major societal and health costs, such that over 75% of the financial burden on the public due to ethanol‐related incidents is associated with binge consumption (Sacks et al., 2015). Furthermore, repeated cycles of binge drinking can lead to the development of alcohol use disorder (AUD) (Centers for Disease Control and Prevention [CDC], 2010), which is related to several negative health outcomes (Alpert et al., 2024; CDC, 2024; Kirpich et al., 2017; Nolen‐Hoeksema, 2004). While AUD diagnoses have historically been more common in men than in women, not only has the number of females diagnosed with AUD risen in recent years (Keyes et al., 2008), but women also experience faster AUD and related health risk progression (White, 2020). Furthermore, negative affect and stressful life events have been strongly associated with the onset of AUD in women, making them more likely to develop an AUD resulting from stress than males (Verplaetse et al., 2018). Women are more likely to relapse in response to a stressful event, as well as being sensitized to stress during periods of withdrawal (Becker & Koob, 2016; Peltier et al., 2019; Sharrett‐Field et al., 2013). Taken together, these data show a sexually dimorphic pattern of interactions of stress and alcohol consumption in males and females. However, in preclinical research, the underlying neural mechanisms that modulate these sex differences are almost entirely unexplored.

Neuropeptide Y (NPY) is expressed at high levels in brain regions regulating affective behavior, energy homeostasis, and memory, such as the amygdala, hypothalamus, hippocampus, and locus coeruleus (Brothers & Wahlestedt, 2010; Kask et al., 2002; Kautz et al., 2017). In these regions, NPY is thought to provide stress resilience by counteracting corticotropin releasing factor (CRF)‐mediated stress particularly via Y1 receptors (Eva et al., 2006). In the central nervous system, the Y1R and Y2R are best characterized and are both Gi/o‐coupled receptors that activate several downstream signaling pathways, such as inhibition of cyclic AMP production, regulating calcium release, and activation of G Protein‐Coupled Inwardly‐Rectifying Potassium (GIRK) channels (Harfstrand et al., 1987; Herzog et al., 1992; Palmiter et al., 1998). While the Y1R is typically expressed post‐synaptically and regulates cellular activity, the Y2R functions pre‐synaptically, either as an autoreceptor that regulates NPY release or as a heteroreceptor that can control the release of other neurotransmitters (Chen et al., 1997; Dumont et al., 1993; Gilpin et al., 2011; Greber et al., 1994; Gustafson et al., 1997; Kopp et al., 2002; Parker & Herzog, 1999), such as GABA and glutamate (King et al., 2000; Qian et al., 1997; Smith‐White et al., 2001; Wahlestedt et al., 1986).

NPY's action in both stress and alcohol intake behaviors makes it a key neuromodulator as an “anti‐stress” neuropeptide. Especially in the CeA, the NPY system has been implicated in modulating anxiety‐like behaviors and response to stress (Heilig et al., 1993; Nakajima et al., 1998; Sajdyk et al., 2002), a mechanism that seems to be primarily driven by Y1Rs (Heilig et al., 1993). Similarly, higher levels of anxiety‐like behaviors are associated with lower NPY levels in the amygdala of alcohol‐preferring rats (Möller et al., 1997). Local NPY infusion into the basolateral amygdala (BLA) (Robinson et al., 2023), or CeA (Zhang et al., 2009) also leads to decreases in ethanol consumption in rodents, which shows a consistent interplay of this neuropeptide in the amygdala with reducing both anxiety and ethanol intake. Furthermore, Y1R and Y2R act in opposition, such that a Y1R agonist and a Y2R antagonist both blunted ethanol binge drinking in mice (Eva et al., 2006; Sparrow et al., 2012). These data suggest that both NPY receptor subtypes may work together to modulate the impact of NPY on stress and alcohol‐related behaviors.

While manipulating NPY receptor signaling modulates ethanol intake, it is also the case that ethanol intake induces plasticity in the NPY system. Chronic ethanol consumption induces region‐specific changes in NPY expression with both short‐ and long‐term effects. Excessive alcohol intake has been linked to increased NPY expression and reduced Y1 receptor RNA in the dorsal raphe nucleus (McClintick et al., 2014), while ethanol exposure decreases NPY levels in the nucleus accumbens (Barkley‐Levenson et al., 2016). These effects are dynamic, as ethanol withdrawal initially reduces NPY expression, followed by overexpression after prolonged abstinence (Bison & Crews, 2003; Criado et al., 2011). Our laboratory has observed similar patterns in the central amygdala, where binge‐like ethanol intake reduced NPY and Y1R immunoreactivity, while a 24‐h abstinence period increased Y1R and Y2R levels (Sparrow et al., 2012). These findings suggest that long‐term ethanol exposure alters the NPY system, with partial recovery during abstinence.

As the CeA is the main output area related to behavioral responses to stress, it has strong projections to areas such as the lateral hypothalamus (LH) (Gilpin & Roberto, 2012; Koob, 2008). A CRF1R+ CeA‐to‐LH neurocircuitry has previously been shown to modulate stress response and ethanol self‐administration in male and female rats (Weera et al., 2021, 2023). Furthermore, our laboratory has explored the role of CRF signaling in the CeA‐LH pathway and discovered sex‐specific mechanisms regarding ethanol binge consumption. Our results showed that chemogenetically silencing a CRF+ circuit between the CeA and LH significantly blunts binge‐like ethanol drinking and blood ethanol concentrations (BECs) in male, but not female, mice (Bendrath et al., 2024). Thus, CRF+ CeA projections to the LH modulate both responses to stressful stimuli and ethanol consumption in male mice only.

There is evidence that the NPY system may modulate neurobiological response to ethanol in a sex‐dependent manner. While both male and female NPY 1 receptor (Y1R) knockout mice show similar increases in ethanol consumption and preference over controls (Thiele et al., 2002), local infusion of NPY into the BLA reduces binge‐like ethanol intake in males but not females (Robinson et al., 2023), further supporting sexually dimorphic NPY system function in stress and alcohol‐related behaviors. These findings highlight the need to further explore how sex differences in NPY signaling influence ethanol consumption, particularly in stress‐responsive brain regions like the CeA.

Building on evidence of NPY's modulatory effects on binge‐like ethanol drinking in the CeA and the contributions of a CeA‐to‐LH circuit to this behavior in a sex‐dependent manner, this study assessed the role of Y1R+ CeA‐LH projections in ethanol consumption using chemogenetics and transgenic mouse lines. To examine how enhanced NPY signaling in the CeA influences binge drinking (DID) and intermittent access to ethanol (IAE), a viral vector was designed and used to drive constitutive overexpression and secretion of NPY via a fibronectin (FIB)‐tagged construct (Foti et al., 2007; Haberman et al., 2003; McCown, 2006). Finally, the DID paradigm was used for immunohistochemistry analyses of Y1R protein, as it models binge‐like intake and has been previously used by our laboratory to assess ethanol‐related changes in protein expression (Sparrow et al., 2012). Additionally, Y1R expression was quantified in NeuN+ neurons to assess ethanol‐induced Y1R plasticity with cell‐type specificity.

## MATERIALS AND METHODS

### Overall experimental design

There were three experiments performed: Experiment 1 focused on the chemogenetic manipulation of CeA‐to‐LH Y1R+ projections in NPY1R‐cre mice. Experiment 2 used a viral NPY overexpression vector (FIB‐NPY) to assess the effect of NPY signaling in the CeA of male and female mice. Experiment 3 assessed Y1R protein expression in the CeA of male and female mice after 6 weeks of ethanol DID or water consumption. A summary of procedures is presented below; please see supplemental material for further details.

### Animals

Details on animals are outlined in the supplemental methods. Briefly, Experiment 1 used 37 male and female NPY1R‐ires‐Cre (Y1R‐Cre) mice. Experiment 2 used 34 NPY1R‐cre negative animals FIB‐NPY: male, *n* = 17; female, *n* = 17; Control: male, *n* = 16; female, *n* = 15. Experiment 3 used 30 male and 30 female C57BL/6 J mice (Jackson Laboratories, Bar Harbor, ME).

### Surgery

All surgeries were conducted on an Angle II stereotax (Leica Instruments, Buffalo Grove, IL). For Experiment 1, mice received either a Cre‐dependent control vector (AAV8‐hSyn‐DIO‐mCherry, Catalog #: 50459‐AAV8, Addgene, Watertown, MA; *n* = 21) or the Cre‐dependent Gi/o‐coupled DREADD vector (AAV8‐hSyn‐DIO‐hM4D(Gi)‐mCherry, Catalog #: 44362‐AAV8, Addgene, Watertown, MA; *n* = 16) into the central amygdala at (AP: −1.06, ML: ±2.42, DV: −4.63). For Experiment 2, mice received a bilateral microinfusion of either the AAV‐control vector containing the chicken beta‐actin promoter (CBA) (rAAV2‐ssCBA‐GFP; titer: 2.4 × 10^12^ vg/mL; UNC Vector Core) or the AAV‐construct containing the laminar protein fibronectin bound to the full NPY peptide (FIB‐NPY) overexpression vector (rAAV2‐CBA‐FIB‐NPY; titer: 6.1 × 10^12^ vg/mL; UNC Vector Core) into the central amygdala (AP: −1.06, ML: ±2.42, DV: −4.63). Both viral constructs were provided by Dr. Thomas McCown and replicated at the UNC Vector Core.

### Drinking studies

“Drinking in the Dark” (DID) and two‐bottle IAE procedures are outlined in detail in the supplemental methods. Briefly, the DID study followed standard protocol as outlined previously (Thiele & Navarro, 2014). For FIB‐NPY animals, after completing 3 weeks of DID, the effect of NPY overexpression and secretion was assessed using the chronic IAE model, which has previously been shown to promote dependence‐like phenotypes (Hwa et al., 2011).

### Open Field

Open‐field testing was used to determine whether viral‐mediated overexpression of NPY impacted animals' locomotor or anxiety‐like behaviors following DID and IAE testing.

### Drug administration

For Experiment 1, approximately 30 min prior to ethanol access on the 4th day of DID, animals received microinjections with either the DREADD activator, clozapine‐N‐oxide (CNO; 99 pmol) or vehicle (1% DMSO in 0.9% saline), in a counterbalanced 2x2 Latin‐square design during consecutive weekly DID sessions. Drug injections were performed using a Hamilton syringe (Reno, NV) attached to a Harvard Apparatus PHD 2000 infusion pump (Holliston, MS) at a rate of 1 μL for 3 min (3 μL total). Infusion needles stayed in the cannula for an additional minute to ensure complete diffusion. Tail blood samples were collected by nicking the lateral tail vein on the 4th day immediately after ethanol access (about 30 μL) to assess BECs on an (AM1) Alcohol Analyzer (Analox, London, UK).

### Perfusion and histology

For cannula placement checks, DREADD expression, and IHC, mice were perfused, and tissue was sectioned on a vibratome (Leica VT1000S vibratome; Wetzlar, Germany). Tissue was then mounted for imaging or stored for immunohistochemical staining.

### Tissue collection and preparation (for Experiment 2)

Once FIB‐NPY mice had concluded DID, IAE, and open‐field testing, animals were euthanized via rapid decapitation for brain extraction and processing of brain tissue for RT‐qPCR.

### RNA extraction, cDNA synthesis, and RT‐qPCR (for Experiment 2)

The protocols for RNA extraction, cDNA synthesis, and RT‐qPCR were based on methods described previously (Barkell et al., 2022; Dankert et al., 2025), and can be found in the supplemental section.

### Immunohistochemistry

In short, CeA brain slices were processed for immunohistochemistry using primary antibodies against Y1R (1:500) (Ref No.: PA5‐102698; Invitrogen, Rockford, IL). Western blot analyses were performed by the vendor to assess the specificity of the antibody to the Y1R (see https://www.thermofisher.com/antibody/product/NPY1R‐Antibody‐Polyclonal/PA5‐102698) and NeuN (1:500) (Ref. No.: PA5‐143586; Invitrogen, Rockford, IL), followed by incubation with fluorescent secondary antibodies (Alexa Fluor™ 488 (1:1000), Ref. No: A‐21206; Invitrogen, Rockford, IL. Alexa Fluor™ 647 (1:1000), Ref. No: A‐21447; Invitrogen, Rockford, IL). Tissue was mounted and cover slipped after thorough rinsing, with control sections processed without primary antibody to confirm specificity.

### Confocal microscopy and image processing (for Experiment 3)

In short, confocal images were acquired using a Zeiss LSM800 microscope, processed with AutoQuant X3 (MediaCybernetics) for deconvolution, and analyzed in Imaris (Zurich, Switzerland) for colocalization. NPY1R colocalization with NeuN was quantified as the percentage of Y1R signal volume (above background threshold) that overlapped with NeuN signal, representing the extent of Y1R expression within neurons from six bilateral Z‐stacks per animal, with outliers identified using Grubb's test. The number of Y1R‐expressing neurons was not quantified, as Y1R immunoreactivity was consistently detected in the vast majority of NeuN+ cells across all animals, with no apparent group differences.

### Statistical analysis

All analyses and graphs were generated with GraphPad Prism (GraphPad Software, Inc. La Jolla, CA, USA), and SPSS (IBM Analytics, Armonk, New York). Outliers within each group were identified and removed prior to analysis using Grubbs' Test. Unpaired two‐tailed t‐tests or repeated measures analysis of variance (ANOVA) were used to assess experimental treatment effects between male and female mice on ethanol intake and BEC's. For the IHC experiment, a two‐way ANOVA was used to assess the effects of sex and DID group on NPY1R expression. A three‐way ANOVA was used to determine the effects of time, sex, and drinking group on body weight. To determine relationships of sex, DID group, and NPY1R expression, simple linear regressions and correlations were conducted. For the FIB‐NPY experiment, if no sex differences were detected, data were collapsed across sex. qPCR data were analyzed using the comparative CT (∆∆CT) method. Housekeeper beta‐actin expression was calculated using the 2^−ΔCT^ method, and values were expressed as a percentage of the average across all samples to evaluate stability across groups. Then, target gene expression in each sample was initially normalized to the housekeeping gene (beta actin), followed by normalization to the mean expression level across all samples. Simple linear regressions were used to determine the relationships between FIB‐NPY, Y1R, and Y2R mRNA expression with weekly averages of DID or IAE consumption. Data were considered significant at *p* < 0.05 (two‐tailed), and a Tukey's post hoc test was used for significant ANOVA effects, as well as planned comparisons.

## RESULTS

### Experiment 1: Gi DREADD inhibition of Y1R+ CeA‐LH circuit reduced binge‐like ethanol intake in male mice only

**FIGURE 1:**
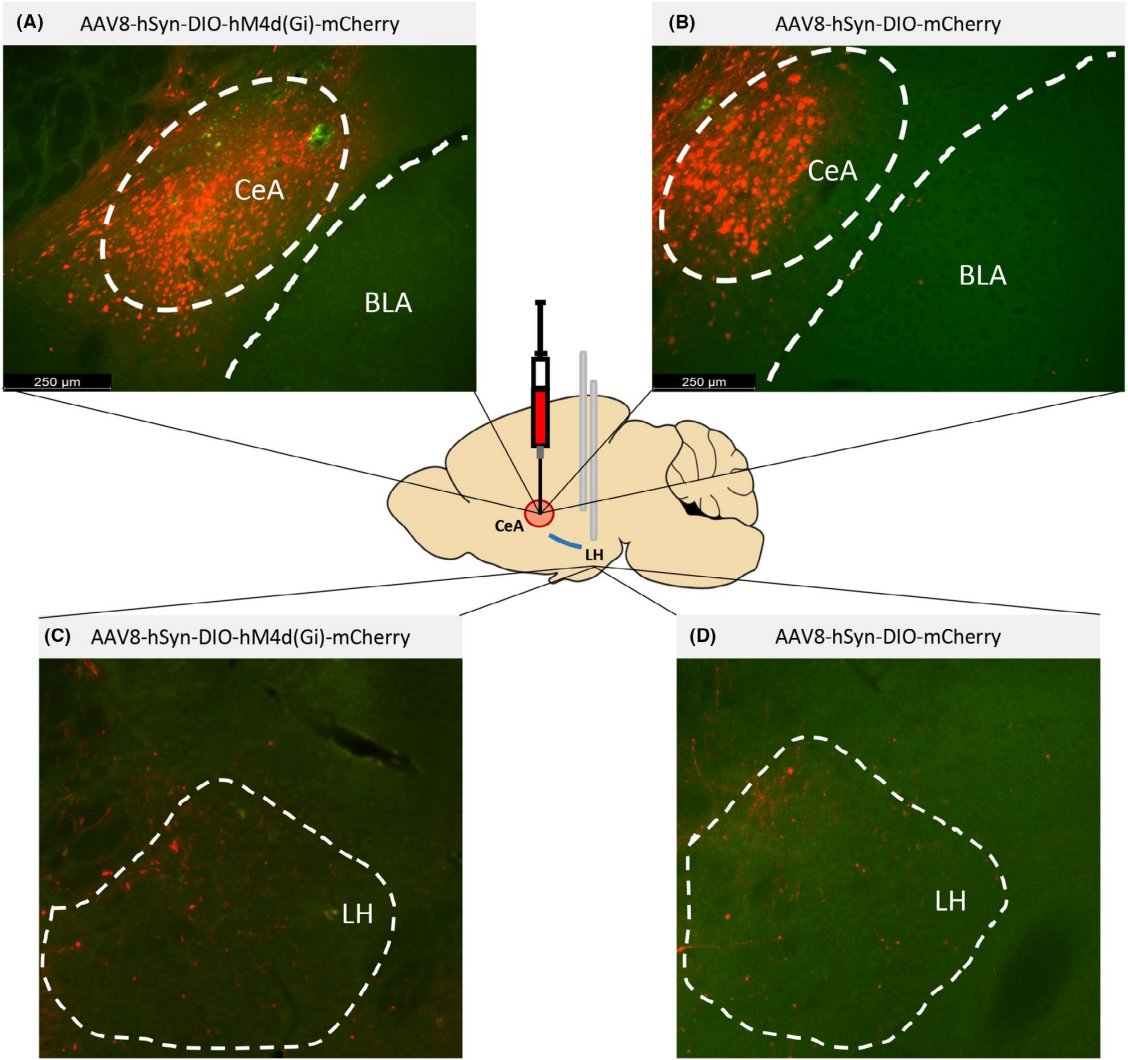
Photomicrographs showing AAV8‐hSyn‐DIO‐hM4d(Gi)‐mCherry virus expression in the CeA (A) and AAV8‐hSyn‐DIO‐mCherry control virus expression in the CeA (B). LH terminals for hM4D(Gi) (C) and mCherry control images (D) of NPY1R‐ires‐cre mice. The central schematic shows virus infusion into the CeA and cannula placement into the LH. BLA, basolateral amygdala; CeA, central nucleus of the amygdala; LH, lateral hypothalamus.

**FIGURE 2:**
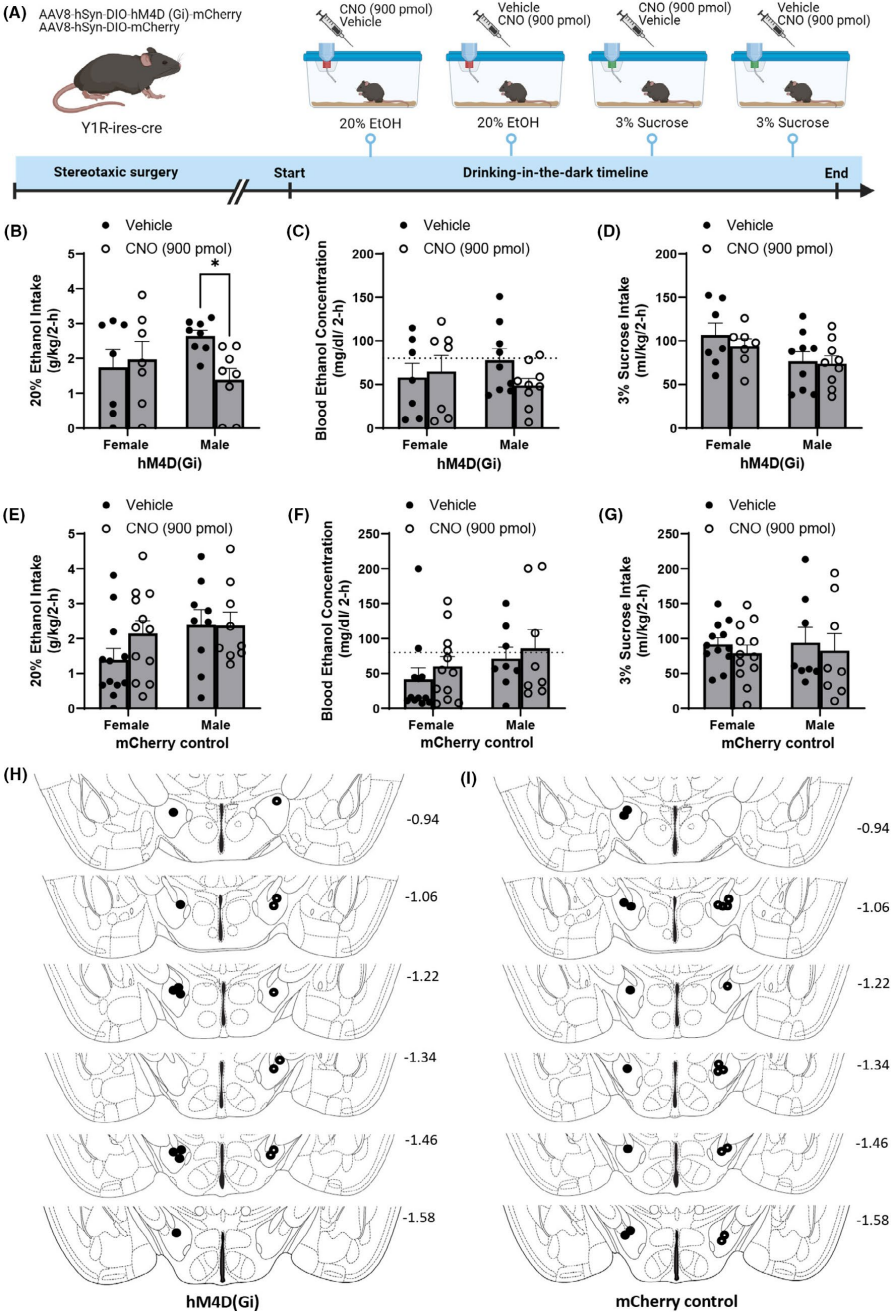
Chemogenetic silencing of the Y1R+ CeA‐LH circuit and control virus experiments. (A) Timeline of procedures used in the chemogenetic experiments (Created with BioRender®). (B) Only male mice show a significant reduction in binge‐like ethanol intake when the Y1R+ CeA‐LH circuitry is inhibited. No significant changes are observed in BECs (dotted line indicates 80 mg/dL) (C) or sucrose intake (D) based on CNO/Veh treatment. (E–G) No significant changes are seen in (E) ethanol consumption, (F) BECs (dotted line indicates 80 mg/dL), (G) or sucrose intake in male and female mice depending on vehicle or CNO treatment. (H, I) Cannula placement for hM4d(Gi)‐DREADD (H) or control (I) animals; placements are marked to the left (filled circles) for male and to the right (open circles) for female animals. Due to the cannula being placed within a pedestal, only 1 hemisphere is shown for placement because each subject was consistent across hemispheres. Data are represented as mean ± SEM. *p < 0.05. CNO, clozapine‐N‐oxide; NPY, Neuropeptide Y.

Viral Gi‐ and control mCherry expression in the CeA can be seen in Figure 1A,B. Figure 1C,D depict LH terminal expression. Figure 2A shows a schematic overview of procedures used in Experiment 1. Our data indicate that there was a pattern of sex‐specific reduction in ethanol consumption in male, but not female mice expressing the Gi DREADD virus (hM4D(Gi)), when CNO was injected and the Y1R+ CeA ➔ LH pathway was inhibited (Sex: *F*(1,13) = 0.107, *p* = 0.75; Injection: *F*(1,13) = 3.112, *p* = 0.10; Sex × Injection: *F*(1,13) = 6.483, *p* = 0.02) relative to vehicle treatment (Figure 2B). Planned comparisons indicate that males significantly reduced their ethanol consumption after CNO infusion (*p* = 0.015), but not females (*p* = 0.841). No such sex‐specific changes were seen in BEC values (Sex: *F*(1,14) = 0.017, *p* = 0.90; Injection: *F*(1,14) = 0.673, *p* = 0.426; Sex × Injection: *F*(1,14) = 1.787, *p* = 0.203) (Figure 2C). Likewise, there was no significant change in sucrose consumption in either hM4D(Gi) males or females when treated with CNO or vehicle (Sex: *F*(1,14) = 3.221, *p* = 0.094; Injection: *F*(1,14) = 1.400, *p* = 0.257; Sex × Injection: *F*(1,14) = 0.587, *p* = 0.257) (Figure 2D).

Control mCherry virus expressing NPY1R‐cre mice exhibited no change in ethanol consummatory behavior when infused with CNO versus vehicle (Sex: *F*(1,19) = 2.241, *p* = 0.151; Injection: *F*(1,19) = 1.277, *p* = 0.273; Sex × Injection: *F*(1,19) = 1.401, *p* = 0.251) (Figure 2E). Likewise, BECs remained stable across sex and treatment conditions (Sex: *F*(1,18) = 1.489, *p* = 0.238; Injection: *F*(1,18) = 1.755, *p* = 0.202; Sex × Injection: *F*(1,18) = 0.021, *p* = 0.888) (Figure 2F). Sucrose consumption also did not vary among mCherry control virus animals when considering sex and treatment received (Sex: *F*(1,18) = 0.020, *p* = 0.890; Injection: *F*(1,18) = 2.949, *p* = 0.103; Sex × Injection: *F*(1,18) = 0.014, *p* = 0.908) (Figure 2G). Cannula placement for hM4d(Gi)‐DREADD (Figure 2H) or control (Figure 2I) animals is shown, with placements marked to the left (filled circles) for male, and to the right (open circles) for female animals.

### Experiment 2.1: CeA NPY Viral overexpression and secretion did not alter ethanol intake, but did affect locomotor behavior

**FIGURE 3:**
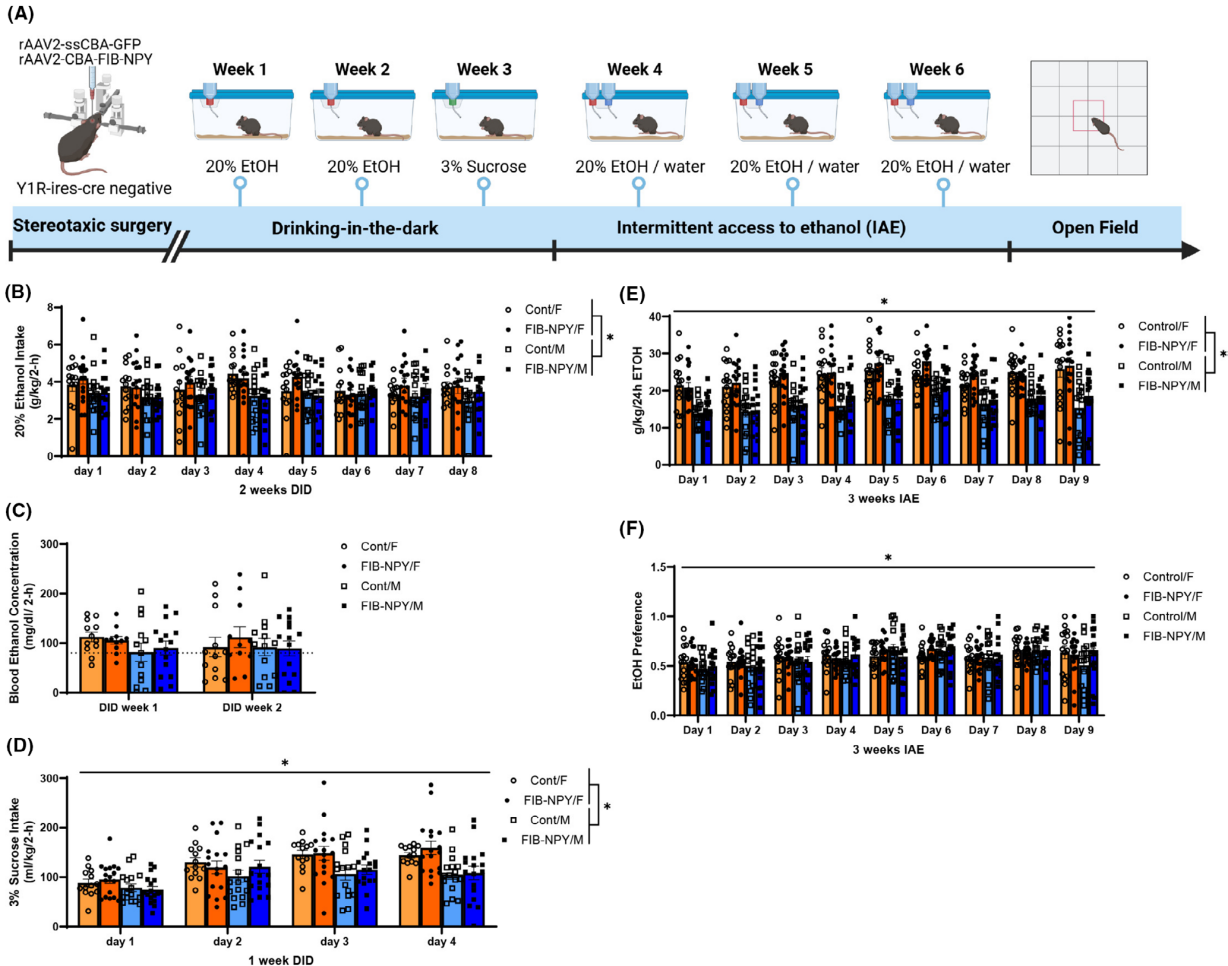
DID and IAE data for male and female mice treated with either FIB‐NPY (NPY overexpression) or control virus. (A) Timeline of procedures used in the NPY overexpression experiments (Created with BioRender®). (B) DID ethanol consumption was overall higher in females than males, but there were no differences in ethanol consumption based on virus condition (C). Similarly, BEC's were not affected by either virus manipulation in female and male mice (dotted line indicates 80 mg/dL). (D) Sucrose consumption shows differences between DID days for control and FIB‐NPY female and FIB‐NPY males, but these differences were never between virus groups, only within. (E) IAE ethanol consumption varied across days, and females overall consumed more than males, but ethanol intake did not significantly vary between virus groups on any day. (F) Ethanol preference did not vary by sex but varied across time. Data are represented as mean ± SEM. *p < 0.05. BEC, blood ethanol concentration; CeA, central nucleus of the amygdala; DID, drinking in the dark; IAE, intermittent access to ethanol; NPY, neuropeptide Y; Y1R, neuropeptide Y receptor 1.

A schematic showing the experimental timeline is represented in Figure 3A. DID analyses were done across sex and virus to assess animals' ethanol consumption for 2 weeks, BECs after the last day of drinking each week, and sucrose consumption for 1 week. If an animal had a missing value or leaked bottle on one DID day, it was excluded from the analysis. DID ethanol consumption did not significantly vary across time (*F*(7, 392) = 0.8381, *p* = 0.5562) or virus (*F*(1, 56) = 0.7755, *p* = 0.3823), but there was a significant main effect of sex (*F*(1, 56) = 5.61, *p* = 0.0213), showing that females overall consumed more ethanol than males. The interactions of time by sex (*F*(7, 392) = 1.795, *p* = 0.0869), time by virus (*F*(7, 392) = 0.6703, *p* = 0.6973), sex by virus (*F*(1, 56) = 0.0065, *p* = 0.9362), and the three‐way interaction of time, sex, and virus (*F*(7, 392) = 0.7649, *p* = 0.6171) all were not significant (Figure 3B). BECs did not significantly vary across time (*F*(1, 47) = 0.0147, *p* = 0.904), sex (*F*(1, 47) = 2.231, *p* = 0.1419), and virus group (*F*(1, 47) = 0.1654, *p* = 0.6861). Similarly, the interactions of time × sex (*F*(1, 47) = 0.2834, *p* = 0.597), time × virus (*F*(1, 47) = 0.1163, *p* = 0.7346), sex × virus (*F*(1, 47) = 0.0251, *p* = 0.8748), and a three‐way interaction of time, sex, and virus (*F*(1, 47) = 0.7058, *p* = 0.4051) were all not significant (Figure 3C).

Finally, one DID week of sucrose consumption significantly varied across time (*F*(3, 177) = 33.80, *p* < 0.0001), and sex (*F*(1, 59) = 9.175, *p* = 0.0036), but virus was not a significant main effect (*F*(1, 59) = 0.3140, *p* = 0.5773). The interaction of time and sex was significant (*F*(3, 177) = 4.508, *p* = 0.0045), indicating that sucrose consumption varied across time by sex, with female mice generally consuming more sucrose than males. Finally, the interaction of time by virus (*F*(3, 177) = 0.1683, *p* = 0.9177), sex by virus (*F*(1, 59) = 0.034, *p* = 0.8543), and three‐way interaction of all variables (*F*(3, 177) = 1.745, *p* = 0.1595) did not significantly affect sucrose consumption (Figure 3D).

As DID consumption only captures a short binge‐drinking window of ethanol intake, but the intra‐CeA injection of the FIB‐NPY viral construct represents a constitutive change in NPY signaling, animals also went through 3 weeks of IAE. Twenty‐four‐hour ethanol consumption across 3 weeks of IAE was significantly affected by time (*F*(8, 488) = 8.966, *p* < 0.0001), and sex (*F*(1, 61) = 65.45, *p* < 0.0001), indicating that overall, animals increased the amount of ethanol they consumed, and females overall consumed more ethanol than males. However, ethanol intake did not significantly vary between virus groups (*F*(1, 61) = 1.878, *p* = 0.1756). Furthermore, the interactions of time by sex (*F*(8, 488) = 0.9042, *p* = 0.5126), time by virus (*F*(8, 488) = 0.6995, *p* = 0.6921), and sex by virus (*F*(1, 61) = 6.892e‐006, *p* = 0.9979) were all not significant. A three‐way interaction of time, sex, and virus (*F*(8, 488) = 0.5548, *p* = 0.8148) also did not significantly alter ethanol intake (Figure 3E).

Ethanol preference was computed by dividing the amount of ethanol consumed by the total amount of fluid consumed across a 24‐h period. Ethanol preference varied significantly by day (*F*(8, 488) = 8.911, *p* < 0.0001), such that there was a slight increase in preference over the three IAE weeks. However, ethanol preference was not significantly different between male and female mice (*F*(1, 61) = 0.5919, *p* = 0.4447), nor between FIB‐NPY and control treated mice (*F*(1, 61) = 0.1974, *p* = 0.6584). Furthermore, the interactions of time by sex (*F*(8, 488) = 1.009, *p* = 0.4281), time by virus (*F*(8, 488) = 0.4452, *p* = 0.8936), and sex by virus (*F*(1, 61) = 0.0636, *p* = 0.8017) were all not significant. A three‐way interaction of time, sex, and virus (*F*(8, 488) = 1.268, *p* = 0.258) also did not significantly alter ethanol intake (Figure 3F).

**FIGURE 4:**
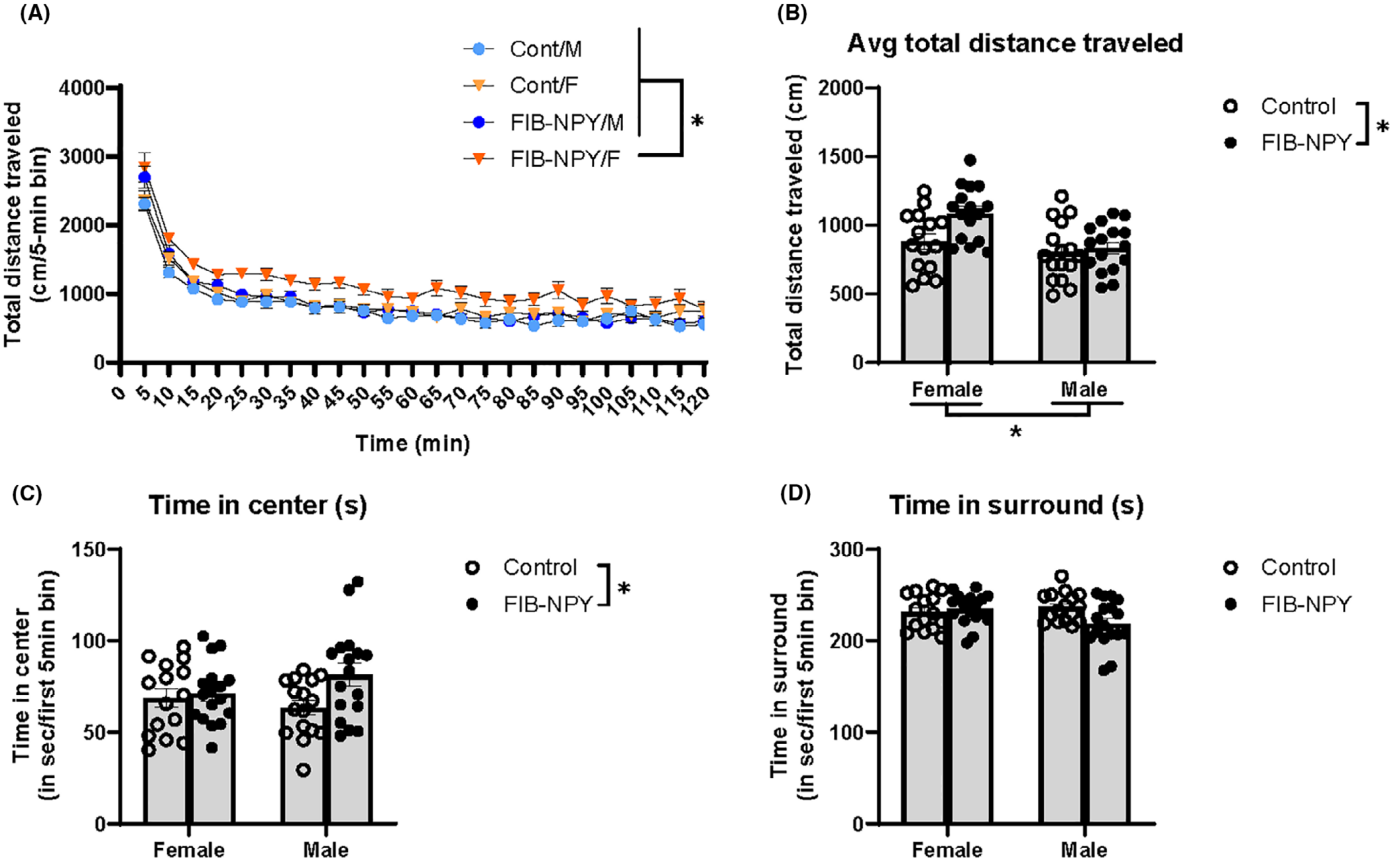
Open‐field test behavior of FIB‐NPY and control mice. (A) Across the 2‐h time spent in the open‐field box, all animals decreased their locomotion significantly, with female FIB‐NPY mice moving more overall than the others. (B) Comparing average distance traveled in the open field, again FIB‐NPY females moved significantly more than control females, or FIB‐NPY and control males. (C) While there was no significant sex by virus interaction, FIB‐NPY mice spent significantly more time in the center than control mice. (D) There were no differences between sex or virus group in the time animals spent on the surrounding edges of the chamber. Data are represented as mean ± SEM. *p < 0.05.

To assess whether the FIB‐NPY virus affects not just drinking behavior but also locomotion and anxiety‐like behaviors (similar to what was observed by Thorsell et al., 2007), animals were run through a 2‐h period in the open‐field test following IAE testing. A three‐way ANOVA was run to capture the effects of time, virus treatment, and sex on locomotor behavior. There were significant main effects of time (*F*(23, 1403) = 166.9, *p* < 0.0001), virus (*F*(1, 61) = 7.58, *p* = 0.0078), and sex (*F*(1, 61) = 7.982, *p* = 0.0064), as well as a significant interaction of time and virus (*F*(23, 1403) = 1.925, *p* = 0.0054). The interactions of time and sex (*F*(23, 1403) = 0.7728, *p* = 0.7686), virus and sex (*F*(1, 61) = 2.411, *p* = 0.1256), as well as the three‐way interaction of time, virus, and sex (*F*(23, 1403) = 0.7272, *p* = 0.8212) all did not significantly impact locomotor behavior (Figure 4A). Average distance traveled (Figure 4B) was affected by sex (*F*(1, 59) = 10.73, *p* = 0.0018) and virus (*F*(1, 59) = 5.63, *p* = 0.0209), but not an interaction of the two (*F*(1, 59) = 2.979, *p* = 0.0896).

To assess anxiety‐like behavior (with more time spent in the center of the locomotor chamber reflecting reduced anxiety‐like behavior), the first 5 min in the open‐field test were assessed with respect to the time spent in the center of the chamber vs. the surrounding edges. Sex did not significantly affect center time (*F*(1, 61) = 0.2706, *p* = 0.6048), while virus condition did (*F*(1, 61) = 4.453, *p* = 0.0389). An interaction of sex and virus also did not impact time spent in the center (*F*(1, 61) = 2.543, *p* = 0.116) (Figure 4C). For surround time, there were no significant effects of sex (*F*(1, 61) = 1.254, *p* = 0.2671) or virus (*F*(1, 61) = 2.254, *p* = 0.1384), but an interaction of the two variables had a significant impact on time spent in the surround (*F*(1, 61) = 4.8, *p* = 0.0323) (Figure 4D).

### Experiment 2.2: qPCR mRNA Expression of Y1R and Y2R does not significantly vary between control and FIB‐NPY animals

**FIGURE 5:**
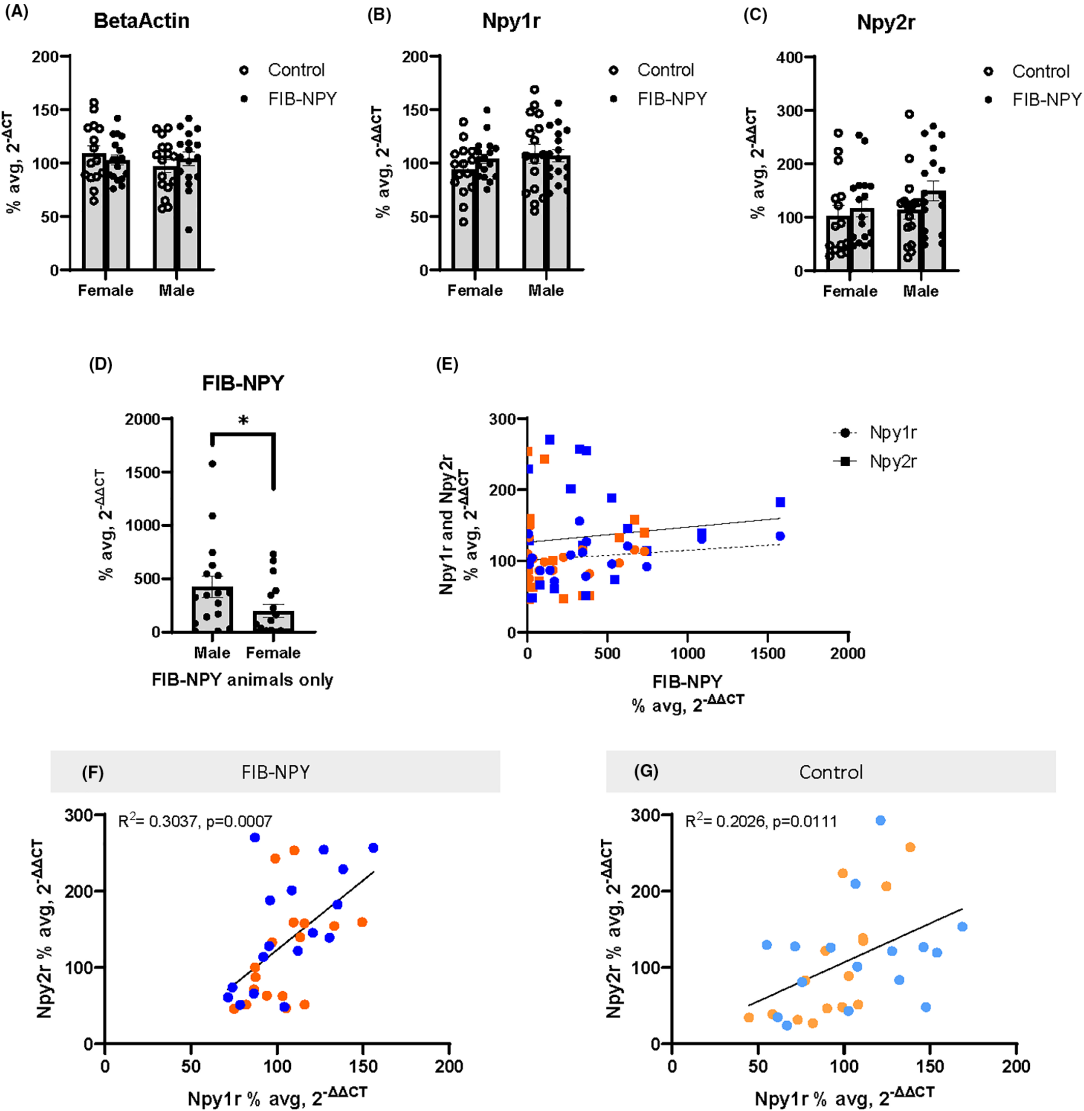
qPCR results of beta actin, Y1R, Y2R, and FIB‐NPY virus mRNA expression in amygdala punches. (A) Beta actin, (B) Y1R, and (C) Y2R mRNA expression do not vary by sex or virus group. (D) Overall, FIB‐NPY mRNA expression was higher in males than in females. (E) There was no significant relationship between the amount of FIB‐NPY virus expressed and Y1R or Y2R mRNA expression. (F) Y1R and Y2R mRNA expression show a significant positive relationship for both FIB‐NPY (F) and control mice (G). Data are represented as mean ± SEM. *p < 0.05. NPY, neuropeptide Y; Y1R, neuropeptide Y receptor 1; Y2R, neuropeptide Y receptor 2.

The four targets run on qPCR were beta actin as the housekeeper control, Y1R, Y2R, and FIB‐NPY (to assess whether the virus was expressed or not). Beta‐actin mRNA expression was not significantly impacted by sex (*F*(1, 61) = 0.9397, *p* = 0.3362), virus (*F*(1, 61) = 0.02313, *p* = 0.8796), or an interaction of sex and virus (*F*(1, 61) = 0.6831, *p* = 0.4117) (Figure 5A). Y1R mRNA expression also did not significantly vary based on sex (*F*(1, 61) = 1.13, *p* = 0.2919), virus (*F*(1, 61) = 1.013, *p* = 0.3182), or an interaction of sex and virus (*F*(1, 61) = 0.6584, *p* = 0.4203) (Figure 5B). Y2R mRNA expression of FIB‐NPY animals did not pass the Shapiro–Wilk test of normality (*W*(34) = 0.92, *p* = 0.018), so a two‐way ANOVA with bootstrapping (1000 samples) was conducted to provide robust estimates of the main effects and interaction term. The main effects of sex (*F*(1, 61) = 1.91, *p* = 0.172, η^2^p = 0.030), and virus (*F*(1, 61) = 2.91, *p* = 0.093, η^2^p = 0.045), as well as an interaction of the two did not significantly affect Y2R mRNA expression (*F*(1, 61) = 0.04, *p* = 0.842, η^2^p = 0.001) (Figure 5C). Finally, the expression of FIB‐NPY virus was assessed in animals. Since this target specifically detected mRNA containing the viral construct, all control animals (lacking the FIB‐NPY construct) got a value of “undetermined” on this target. On this measure, male FIB‐NPY mRNA expression failed the Shapiro–Wilk test of normality (*W*(17) = 0.888, *p* = 0.0429), so FIB‐NPY male and female mice were assessed using the Mann–Whitney test. The results indicate a significant difference between groups (*U* = 87, *p* = 0.049 (two‐tailed)), suggesting that virus expression levels overall were lower in female as opposed to male FIB‐NPY mice (Figure 5D).

Next, linear regressions were conducted to assess relationships between viral and receptor expression. FIB‐NPY expression levels did not significantly predict either Y1R (*F*(1, 32) = 1.846, *p* = 0.1838; *R* ^*2*^ = 0.0545) or Y2R mRNA expression (*F*(1, 32) = 0.3628, *p* = 0.5512; *R* ^*2*^ = 0.0112) (Figure 5E). However, both for FIB‐NPY (*F*(1, 32) = 13.96, *p* = 0.0007; *R* ^*2*^ = 0.3037) and control animals (*F*(1, 29) = 7.37, *p* = 0.0111; *R* ^*2*^ = 0.2026), there were significant linear relationships between Y1R and Y2R mRNA expression (Figure 5F,G). Moving forward, all linear regressions with drinking data (DID and IAE) are done with Y1R and Y2R mRNA expression, as preliminary analyses indicated that there was no significant impact on any dependent measure with FIB‐NPY virus expression (data not shown here).

### Experiment 2.3: qPCR mRNA Expression of Y1R and Y2R in the amygdala is associated with individual differences in IAE ethanol consumption

**FIGURE 6:**
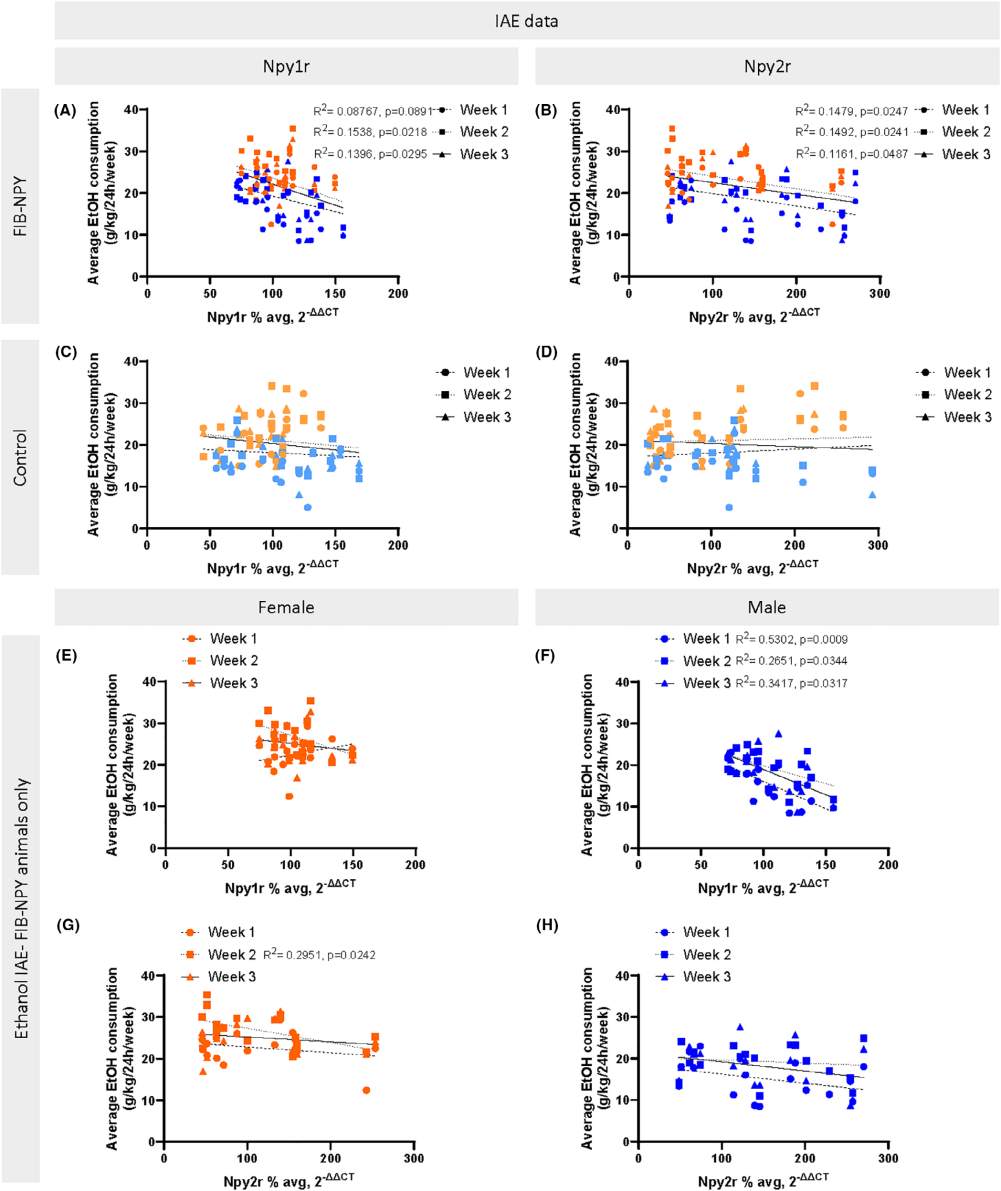
Linear regression of Y1R and Y2R mRNA expression with IAE data and IAE data split by sex. (A) Across 3 weeks of IAE, FIB‐NPY Y1R mRNA expression showed a significant negative linear relationship with Week 2 and 3 ethanol consumption. (B) Higher Y2R mRNA expression in FIB‐NPY animals significantly predicted lower ethanol consumption across all 3 weeks of IAE. For control animals, neither Y1R (C) or Y2R (D) mRNA expression was a significant predictor of ethanol intake across 3 weeks of IAE. (E) Across 3 weeks of IAE ethanol consumption, FIB‐NPY Y1R mRNA expression was not a significant predictor of ethanol intake in female FIB‐NPY mice. (F) However, higher Y1R mRNA expression in FIB‐NPY males significantly predicted lower ethanol consumption across all 3 weeks of IAE. (G) Y2R mRNA expression significantly predicted ethanol consumption only in Week 2 for FIB‐NPY females. (H) In FIB‐NPY males, Y2R mRNA expression showed no significant linear relationship with ethanol consumption across all 3 IAE weeks. Data are represented as mean ± SEM. IAE, intermittent access to ethanol; Y1R, neuropeptide Y receptor 1; Y2R, neuropeptide Y receptor 2.

For all graphics, dark colored data points are FIB‐NPY animals (Figure 6A,B,E–H), and all light‐colored data points are control animals (Figure 6C,D) (light/dark orange = female, light/dark blue = male). All data were collapsed across sex unless there was a significant difference in linear regression when split by sex. As linear regressions with DID consumption and Y1R/Y2R mRNA expression respectively were not significant (Figure S1), the following section focuses on the linear relationship of both receptors with IAE consumption. Linear regressions for Y1R mRNA expression and IAE ethanol consumption for FIB‐NPY animals indicate a trend toward significance Week 1 (*F*(1, 32) = 3.075, *p* = 0.089; *R* ^*2*^ = 0.0877), and a significant negative association between Y1R and IAE Week 2 (*F*(1, 32) = 5.816, *p* = 0.0218; *R* ^*2*^ = 0.1538), as well as Week 3 (*F*(1, 32) = 5.194, *p* = 0.0295; *R* ^*2*^ = 0.1396), indicating that higher Y1R mRNA expression significantly predicted lower ethanol intake (Figure 6A). Y2R mRNA expression in FIB‐NPY animals significantly predicted ethanol intake Weeks 1 (*F*(1, 32) = 5.556, *p* = 0.0247; *R* ^*2*^ = 0.1479), 2 (*F*(1, 32) = 5.611, *p* = 0.0241; *R* ^*2*^ = 0.1492), and 3 (*F*(1, 32) = 4.202, *p* = 0.0487; *R* ^*2*^ = 0.1161) (Figure 6B). Control virus animals' Y1R mRNA expression did not significantly predict ethanol intake across Weeks 1 (*F*(1, 29) = 0.2022, *p* = 0.6563; *R* ^*2*^ = 0.0069), 2 (*F*(1, 29) = 0.694, *p* = 0.4116; *R* ^*2*^ = 0.0234), and 3 (*F*(1, 29) = 0.8986, *p* = 0.351; *R* ^*2*^ = 0.0301) (Figure 6C). Y2R mRNA expression in control mice also had no predictive power for ethanol intake across Weeks 1 (*F*(1, 29) = 0.4287, *p* = 0.5178; *R* ^*2*^ = 0.0146), 2 (*F*(1, 29) = 0.0796, *p* = 0.7798; *R* ^*2*^ = 0.0027), and 3 (*F*(1, 29) = 0.2674, *p* = 0.609; *R* ^*2*^ = 0.0091) (Figure 6D).

Since linear regressions for Y1R and Y2R with IAE ethanol intake were all significant, and given the sex differences observed in the chemogenetic study, these relationships were assessed across sex as well. For FIB‐NPY females, there were no significant relationships between Y1R mRNA and Week 1 (*F*(1, 15) = 1.216, *p* = 0.2876; *R* ^*2*^ = 0.0750), 2 (*F*(1, 15) = 3.276, *p* = 0.0904; *R* ^*2*^ = 0.1793) or 3 (*F*(1, 15) = 3726, *p* = 0.5507; *R* ^*2*^ = 0.0242) ethanol consumption (Figure 6E). However, for FIB‐NPY males, Y1R mRNA expression significantly predicted ethanol consumption for Weeks 1 (*F*(1, 15) = 16.93, *p* = 0.0009; *R* ^*2*^ = 0.5302), 2 (*F*(1, 15) = 5.411, *p* = 0.0344; *R* ^*2*^ = 0.2651), and 3 (*F*(1, 15) = 7.786, *p* = 0.0137; *R* ^*2*^ = 0.3417) (Figure 6F). Thus, for IAE ethanol consumption, the significant linear relationship seen previously (Figure 6A) appears to be driven by male mice. For FIB‐NPY females, Y2R mRNA expression was not a significant predictor of ethanol consumption Week 1 (*F*(1, 15) = 1.006, *p* = 0.3317; *R* ^*2*^ = 0.0629) and Week 3 (*F*(1, 15) = 0.4847, *p* = 0.497; *R* ^*2*^ = 0.0313), but was significant for Week 2 (*F*(1, 15) = 6.278, *p* = 0.0242; *R* ^*2*^ = 0.2951) (Figure 6G). As for FIB‐NPY males, Y2R expression did not show a significant linear relationship to Week 1 (*F*(1, 15) = 2.388, *p* = 0.1431; *R* ^*2*^ = 0.1373), Week 2 (*F*(1, 15) = 0.2614, *p* = 0.6166; *R* ^*2*^ = 0.0171), or Week 3 ethanol intake (*F*(1, 15) = 1.744, *p* = 0.2064; *R* ^*2*^ = 0.1042) (Figure 6H).

### Experiment 3: 6 weeks of DID ethanol consumption did not significantly alter Y1R protein expression, but led to correlation patterns in animals sacrificed immediately after DID or after a 24‐h period of abstinence

**FIGURE 7:**
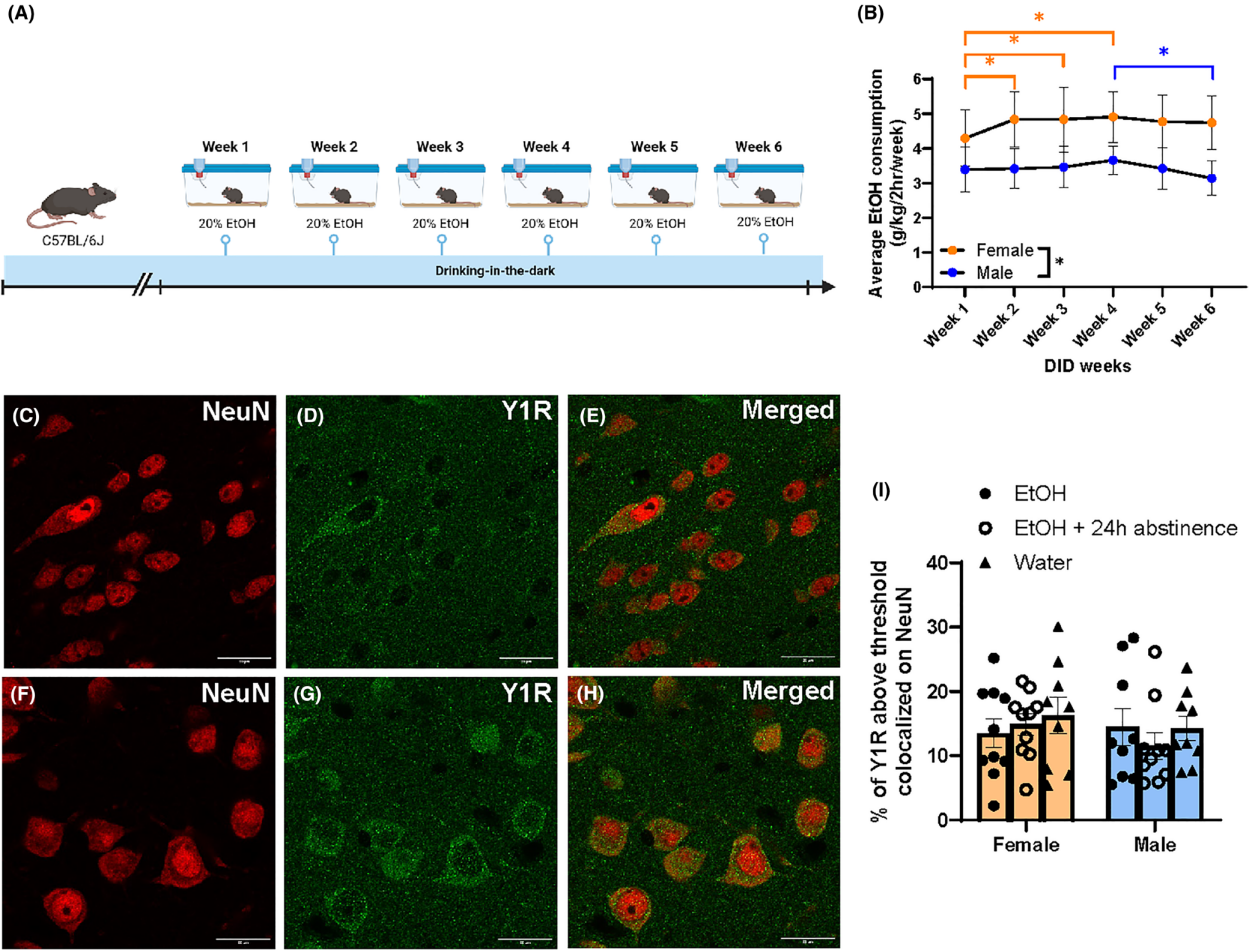
DID paradigm and behavioral analyses. (A) Timeline of procedures used in the IHC DID protocol (Created with BioRender®). (B) Over 6 weeks, females consumed significantly more ethanol than males. Females also increased DID consumption Weeks 2, 3 and 4 compared with Week 1. Males significantly decreased their drinking when comparing Week 4 to Week 6. Representative ethanol‐consuming animal CeA IHC stain showing NeuN (C), Y1R (D), and a merged image of both channels (E). Representative water‐consuming animal CeA IHC stain showing NeuN (F), Y1R (G), and a merged image of both channels (H). (I) % colocalization of Y1R on NeuN in the CeA of male and female mice did not significantly differ based on animals' drinking group. Data are represented as mean ± SEM. *p < 0.05. CeA, central nucleus of the amygdala; DID, drinking in the dark; ETOH, ethanol; IHC, immunohistochemistry; Y1R, neuropeptide Y receptor 1.

For this experiment, animals went through either 6 weeks of ethanol DID, 6 weeks of ethanol DID and 24 h of abstinence, or normal water consumption (Figure 7A). For DID animals, there was a significant main effect of time (*F*(5, 185) = 3.405, *p* = 0.0057), and sex (*F*(1, 37) = 68.81, *p* < 0.0001), but no significant interaction of time and sex on ethanol consumption (*F*(5, 185) = 1.867, *p* = 0.1022) (Figure 7B). A depiction of the IHC stain can be seen in Figure 7, showing NeuN, Y1R, and an overlay for a representative ethanol drinking (Figure 7C–E) and water‐drinking animal (Figure 7F–H). Sex (*F*(1, 51) = 0.6401, *p* = 0.4274), drinking group (*F*(2,51) = 0.4007, *p* = 0.672), and an interaction of sex by drinking group (*F*(2, 51) = 0.4872, *p* = 0.6172) all did not significantly affect Y1R volume colocalization with NeuN in the CeA (Figure 7I).

**FIGURE 8:**
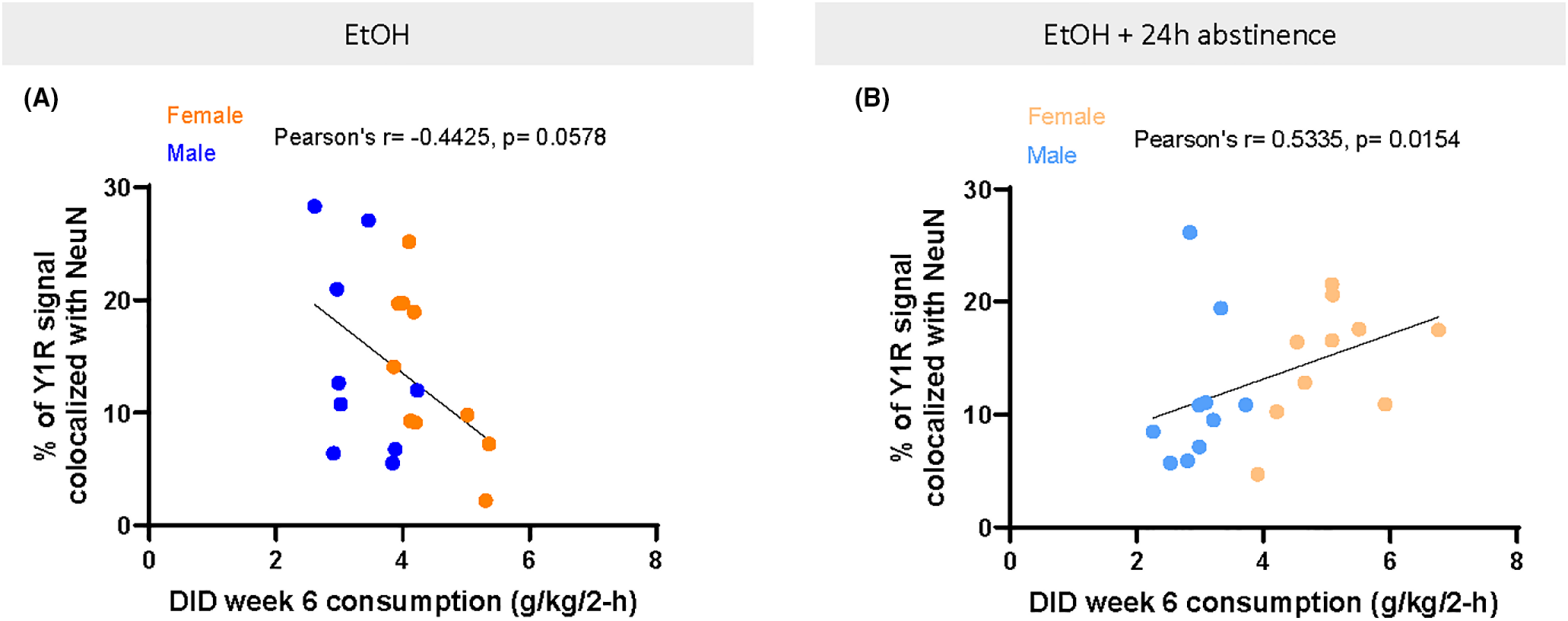
DID drinking behavior correlated with Y1R/NeuN colocalization percentage for ethanol (A) and ethanol with 24‐h abstinence animals (B). There was a trend toward a negative correlation between final week DID consumption and Y1R expression for ethanol mice (A). There was a significant positive correlation between final week DID consumption and Y1R expression for animals that drank ethanol and went through a 24‐h abstinence period (B). Data are represented as mean ± SEM. . DID, drinking in the dark. EtOH, ethanol; Y1R, neuropeptide Y receptor 1.

As no differences were seen in Y1R/NeuN colocalization between sexes, data were collapsed by sex and grouped based on animals being perfused immediately after the final DID session, or after a 24‐h abstinence period. Linear regressions were performed using alcohol consumption from the final DID week (Week 6), as it is closest to perfusion and immunohistochemistry and provides a more reliable measure by averaging intake across all four drinking days. There was a trend toward an effect between Y1R expression and final week DID ethanol consumption (*F*(1, 17) = 4.138, *p* = 0.0578; *R* ^*2*^ = 0.1958) for ethanol animals, as well as a trend toward an effect for 24 h abstinence animals (*F*(1, 18) = 3.808, *p* = 0.0668; *R* ^*2*^ = 0.1746). Since linear regression did not reach significance in this experiment (unlike Experiment 2 analyses), Pearson's *r* is also reported to better capture the strength of association between Y1R expression and final DID week. While regression assesses predictive value, correlation highlights the direction and consistency of the relationship without the same model constraints. This approach is intended to increase transparency and account for potential power limitations in the current sample. Final week DID consumption was again trending toward a negative correlation with Y1R expression for ethanol animals (*r*(18) = −0.4425, *p* = 0.0578) (Figure 8A), and significantly positively correlated with ethanol and abstinence animals (*r*(19) = 0.5335, *p* = 0.0154) (Figure 8B).

## DISCUSSION

Together, these findings demonstrate the complex role of Y1R signaling within the CeA and its projections to the LH in modulating ethanol consumption, highlighting notable sex differences in this regulation. Chemogenetic inhibition of Y1R+ CeA–LH projections reduced binge‐like ethanol drinking in male mice without affecting females or influencing sucrose intake, suggesting a sex‐specific role for this circuit in ethanol consumption. Expanding on this, dynamic interactions between Y1R and Y2R expression in the CeA were observed in relation to ethanol intake, such that chronic NPY overexpression in the CeA via a viral vector produced behavioral effects and predictive relationships between receptor mRNA expression and intake. An increase in Y1R and Y2R expression was associated with a decrease in ethanol consumption, with viral NPY overexpression likely mediating these linear relationships. Additionally, the relationship between Y1R and ethanol intake was stronger in males, while the relationship between ethanol and Y2R only emerged across all AIE weeks when sexes were combined, suggesting sexually divergent mechanisms of Y1R and Y2R in the CeA during ethanol consumption. Further, a history of extended binge‐like drinking influenced Y1R protein expression in the CeA, where higher ethanol intake was associated with lower Y1R immunoreactivity immediately after the final week of DID consumption, with this relationship reversing following a 24‐h abstinence period. Collectively, these results support a model in which Y1R signaling within the CeA contributes to ethanol intake through both circuit‐specific mechanisms and broader neuroadaptive changes, influenced by sex and individual consumption patterns.

Our findings demonstrate that chemogenetic inhibition of a Y1R+ circuit between the CeA and LH significantly reduces binge‐like ethanol drinking in male mice, but has no effect in females (Figure 2B). No changes were observed in sucrose intake in either sex (Figure 2D). Crucially, CNO administration did not alter ethanol consumption, BECs, or sucrose intake in mice expressing the control viral vector (Figure 2E–G). This shows that the observed effects cannot be attributed to off‐target CNO actions (Gomez et al., 2017), as CNO alone had no significant influence on ethanol consumption. This work extends our prior findings of a CRF+ neurocircuitry between the CeA and LH to a Y1R+ circuit (Bendrath et al., 2024), and shows that the neurocircuitry identified here serves as a selective neuronal mechanism in binge‐like ethanol drinking in male mice only. While the current study does not directly examine the overlap between CRF+ and Y1R+ projections from the CeA to the LH, previous work indicates that Y1R signaling can act directly on CRF neurons in the extended amygdala (Pleil et al., 2015). Though additional studies are needed to determine the degree of anatomical convergence, the current findings raise the possibility that Y1R+ and CRF+ projections from the CeA to the LH may represent overlapping or interacting components of the same ethanol‐responsive circuit.

Next, the use of FIB‐NPY overexpression vector provided a unique opportunity to assess both DID and IAE ethanol intake behaviors, as well as receptor mRNA expression through qPCR. Initially, viral NPY overexpression had no significant impact on either DID intake (ethanol and sucrose) or IAE (ethanol, water, and ethanol preference) consumption in either sex (Figure 3). Furthermore, variability in DID consumption was not significantly predicted by Y1R or Y2R mRNA expression in either FIB‐NPY or control animals (Figure S1). A possible explanation is that the constitutive nature of viral NPY overexpression creates a lasting alteration in amygdalar NPY signaling, whereas the short 2‐h binge drinking window may not be sufficient to capture behavioral changes driven by a stable neurobiological modification. It is unlikely that changes in endogenous NPY signaling would have affected drinking behavior. Although endogenous NPY mRNA was not measured, the FIB‐NPY construct drives constitutive production and secretion of NPY, which would be expected to result in elevated NPY signaling regardless of any potential downregulation of endogenous NPY.

While there were no differences in Y1R and Y2R mRNA expression between FIB‐NPY and control animals (Figure 5B,C), Y1R and Y2R mRNA expression was highly predictive of each other in both FIB‐NPY and control mice (Figure 5F,G), suggesting a coordinated regulation of these receptors. This similarity in receptor co‐expression across animals is especially striking given the fact that Y1R and Y2R mRNA expression showed a significant linear relationship with IAE ethanol intake in FIB‐NPY animals, but no such effect in control‐treated mice (Figure 6A–D). Higher Y1R mRNA expression was associated with reduced ethanol intake during Weeks 2 and 3 of IAE, but only in FIB‐NPY‐treated animals (Figure 6A,C), suggesting that these effects were driven by NPY overexpression. The inverse relationship between Y1R mRNA and ethanol consumption aligns with previous findings implicating Y1R in ethanol intake regulation (Sparrow et al., 2012; Thiele et al., 2002) and may reflect a protective role in the CeA similar to its function in other extended amygdala regions (Pleil et al., 2015).

Additionally, Y2R mRNA expression was consistently associated with lower ethanol intake across all three IAE weeks (Figure 6B) only in FIB‐NPY‐treated animals, suggesting again that viral NPY overexpression altered Y2R‐related ethanol modulation. These findings expand on previous research showing reduced Y2R mRNA in alcohol‐preferring rats (Caberlotto et al., 2001), reinforcing Y2R's role in ethanol preference and consumption. While current findings suggest that virus‐induced receptor expression may modulate ethanol intake, the directionality remains unclear. NPY overexpression may either enhance receptor sensitivity to ethanol exposure—leading to greater receptor downregulation in high‐drinking animals—or may induce differential receptor plasticity that, in turn, drives individual differences in drinking behavior. Given prior findings showing reduced ethanol intake after CeA NPY overexpression (Thorsell et al., 2007) and the current findings that CeA‐LH Y1R+ projections modulate ethanol intake, it is plausible that elevated receptor mRNA expression confers protection against ethanol consumption. However, the possibility that drinking levels shape receptor expression cannot be excluded. Together, these results underscore the bidirectional complexity of NPY receptor signaling in the CeA.

Sex differences further shaped these relationships, as the negative correlation between Y1R mRNA and ethanol intake was specific to male mice (Figure 6E,F). This suggests that Y1R mRNA levels are more predictive of drinking behaviors in males than females, potentially due to sex differences in NPY expression and sensitivity (Nahvi & Sabban, 2020). In contrast, Y2R's relationship with ethanol intake was largely consistent across sexes, with only a transient effect in females during Week 2 (Figure 6G,H). Given that viral expression of FIB‐NPY differed between sexes, it is possible that these variations contributed to the observed regression patterns. However, as overall ethanol consumption in both DID and IAE models was not significantly altered by FIB‐NPY treatment in either sex, differences in viral expression alone are unlikely to fully explain the sex‐specific effects. These findings highlight the complexity of NPY‐Y1R/Y2R interactions in ethanol consumption and suggest that future studies should further explore sex‐specific mechanisms underlying these neuropeptide‐driven behaviors.

Finally, current data provide supporting evidence that Y1R expression in the CeA is affected by individual variations in ethanol consumption, such that by the final week of DID consumption, animals with higher ethanol consumption trended toward lower Y1R expression (Figure 8A). Furthermore, this relationship was reversed after a 24‐h period of abstinence, such that there was a significant positive correlation between ethanol consumption and Y1R immunoreactivity (Figure 8B). As these data are correlational, it is possible that extended ethanol exposure in high consumption animals led to reductions in Y1R immunoreactivity, or that inherently lower Y1R expression in some animals was correlated with higher ethanol intake. Yet, the current data support prior findings from our laboratory that showed a reduction in CeA Y1R labeling after multiple cycles of DID, which reversed after a 24‐h abstinence window (Sparrow et al., 2012). A novel contribution of the current experiment is the focus on Y1R plasticity within CeA neurons, achieved by co‐labeling with NeuN to exclude non‐neuronal sources of signal. This approach builds on prior work that lacked cell‐type specificity (Robinson et al., 2023; Sparrow et al., 2012), where DAB staining or fluorescence measures without neuronal markers may have captured Y1R expression across various cell types, including glia. While NeuN is a nuclear marker, it is a widely accepted and specific label for post‐mitotic neurons (Gusel'nikova & Korzhevskiy, 2015). Although it does not capture full cellular morphology, it allowed for more conservative quantification of Y1R immunoreactivity in defined neuronal populations. Given prior findings that Y1R is somatically expressed in both excitatory and inhibitory neurons of the BLA (Rostkowski et al., 2009), it is likely that similar expression patterns are present in the CeA. Thus, in combination with the findings by Sparrow et al. (2012), it seems likely that individual differences in ethanol intake led to reductions in Y1R immunoreactivity in the current experiment. Supporting evidence for this hypothesis also comes from prior findings that the NPY system becomes involved in modulating ethanol intake, particularly in animals that show higher ethanol intake (Badia‐Elder et al., 2001; Thiele & Badia‐Elder, 2003), and that this higher level of intake creates greater neuroadaptation in the NPY/Y1R system. Thus, while current results are only correlational, the pattern of results with ethanol intake, abstinence, and Y1R expression fit into previous literature.

While current results add to the literature on NPY regulating ethanol intake, there are a few limitations to current findings. First, the lack of response in females to chemogenetic inhibition of the Y1R+ CeA‐LH circuitry may indicate that this circuit does not modulate ethanol intake in females, similar to findings in the CRF+ CeA‐LH pathway (Bendrath et al., 2024). However, an alternative explanation is that females may require a higher CNO dose to elicit behavioral changes, as previously observed when females needed a threefold higher dose to reduce binge drinking via BLA‐mPFC inhibition (Robinson et al., 2023). Future studies should investigate whether females are inherently less sensitive to Y1R signaling or whether stronger experimental manipulations are needed to reveal circuit‐specific effects. Second, while viral NPY overexpression altered Y1R and Y2R mRNA expression patterns in relation to IAE ethanol consumption, no relationship emerged for DID consumption. Given that IAE induces higher ethanol intake and more severe withdrawal symptoms than DID (Hwa et al., 2011), it may better capture neuroadaptive changes associated with ethanol dependence, as suggested by previous NPY overexpression studies (Thorsell et al., 2007). Future work should examine FIB‐NPY effects in an extended IAE paradigm to assess its role in high ethanol intake and dependence.

While evidence for sexual dimorphism in the NPY system remains limited, previous studies indicate differential expression of NPY in the amygdala and hypothalamus, with baseline differences in NPY and Y1R expression between male and female rodents that sometimes only emerge following stress (Hill et al., 2014; Miragaia et al., 2018; Nahvi & Sabban, 2020). This raises the possibility that the CeA‐LH circuit influences ethanol consumption in females only when combined with additional stressors, or that distinct Y1R+ CeA circuits differentially modulate binge drinking in males and females. Supporting this, chemogenetic inhibition of the Y1R+ CeA‐to‐lateral habenula circuit decreased binge‐like ethanol consumption in both sexes (Companion et al., 2022), whereas manipulating CRF+ (Bendrath et al., 2024) and Y1R+ projections (Figure 2) between the CeA and LH had no effect in females. Furthermore, while viral NPY overexpression had no significant effect on ethanol, water, or sucrose intake, it produced sex‐specific effects on locomotion and anxiety‐like behaviors by enhancing locomotion in females while reducing anxiety‐like behavior in males. This aligns with previous findings suggesting that males may be more sensitive to NPY's anxiolytic effects (Thorsell et al., 2007), potentially due to generally higher NPY expression (Nahvi & Sabban, 2020). Interestingly, IHC analysis revealed no sex differences in Y1R/NeuN colocalization and no correlation between Y1R expression and ethanol consumption in either sex, despite prior studies indicating ethanol‐related changes in Y1R expression (Robinson et al., 2023; Sparrow et al., 2012). Thus, while the NPY system is influenced by stress and ethanol consumption (Heilig et al., 1993; Möller et al., 1997), the current DID model may not have elicited sex differences comparable to those seen in stress paradigms. Future work should clarify whether females are less sensitive to Y1R signaling in the CeA‐LH circuit or simply require stronger manipulations to reveal sex‐specific effects.

Collectively, these findings provide a unique assessment of NPY signaling during ethanol consumption in the CeA and LH and align with the idea that chronic alcohol use may induce a hypofunctional NPY system, shifting the balance toward CRF‐mediated stress responses, which are known to drive anxiety and relapse behaviors. By integrating molecular, pharmacological, and behavioral approaches, these findings provide valuable insight into the neurobiological mechanisms underlying ethanol consumption and may inform the development of targeted interventions for alcohol‐related disorders.

## CONFLICT OF INTEREST STATEMENT

The authors declare no competing financial interests. Dr. Thiele owns shares of Glauser Life Sciences, a company that aims to develop therapeutics for mental health disorders. The work that is presented in this paper is not directly related to the scientific aims of Glauser Life Sciences.

## Supporting information


Data S1

## Data Availability

The data that support the findings of this study are available from the corresponding author upon reasonable request.
