# Supplementary material for: Neuropeptide Y1 receptor expressing circuit from the central amygdala to lateral hypothalamus modulates binge‐like ethanol consumption in a sex‐dependent manner
Source: Alcohol Clin Exp Res (Hoboken). 2025 Aug 29;49(10):2146–62. doi: 10.1111/acer.70151 (PMC12519052; doi:10.1111/acer.70151)
Supplement: Supplementary file 1 — Data S1 [file ACER-49-2146-s001.docx]

**Supplemental Methods:**

Animals: The in vivo chemogenetic experiment used 37 male and female NPY1R-ires-Cre (Y1R-Cre) mice (positive for the expression of Cre under the NPY1R promoter as determined by standard PCR genotyping protocols). Y1R-Cre mice were 8-10 weeks old at the start of experiment and bred in house (heterozygous X C57BL/6 J (Jackson), originally developed by (Padilla et al., 2016). NPY1R-cre negative animals were used for all FIB-NPY experiments (FIB-NPY: male, n=17; female, n=17; Control: male, n=16; female, n=15). The IHC experiment used 30 male and 30 female C57BL/6 J mice (Jackson Laboratories, Bar Harbor, ME). Upon arrival from Jackson laboratories, animals were given 1 week to acclimate before starting behavioral testing. All mice were housed individually in plastic containers, in the same colony room on a reverse 12 h light-dark cycle, with the lights going off at 0930 hours. Water (unless otherwise stated) and food (Prolab® RMH 3000 (Purina LabDiet®; St. Louis, MO)) were available to all animals ad libitum. All procedures were approved by the University of North Carolina Institutional Animal Care and Use Committee and conducted in accordance with the Guidelines for the Care and Use of Laboratory Animals.

Surgery: Surgeries were conducted on an Angle II stereotax (Leica Instruments, Buffalo Grove, IL). Animals received intraperitoneal (i.p.) injections (1.5 mL/kg) of ketamine (117 mg/kg)/ xylazine (7.92 mg/kg), with all coordinates being measured from bregma. For experiment 1, mice received either a Cre-dependent control vector (AAV8-hSyn-DIO-mCherry, Catalog #: 50459-AAV8, Addgene, Watertown, MA; n=21) or the Cre-dependent Gi/o-coupled DREADD vector (AAV8-hSyn-DIO-hM4D(Gi)-mCherry, Catalog #: 44362-AAV8, Addgene, Watertown, MA; n=16) into the central amygdala at (AP -1.06, ML +/- 2.42, DV -4.63). This Designer Receptor Exclusive Activated by Designer Drugs (DREADD) has been used to manipulate NPY activity in the central amygdala previously (Companion et al., 2022). Additionally, bilateral guide cannulas were implanted into the LH at (AP -1.10, ML +/- 1.10, DV -5.10). Upon surgery completion, all mice recover for at least 3 weeks before starting behavioral testing for maximal viral expression. For experiment 2, mice received a bilateral microinfusion of either the control vector (rAAV2-ssCBA-GFP; titer: 2.4 x 10^12 vg/mL; UNC Vector Core) or the FIB-NPY overexpression vector (rAAV2-CBA-FIB-NPY; titer: 6.1 x 10^12 vg/mL; UNC Vector Core) into the central amygdala (AP -1.06, ML +/- 2.42, DV -4.63). Both viral constructs were provided by Dr. Thomas McCown and replicated at the UNC Vector Core (Haberman et al., 2003, McCown, 2006). Upon surgery completion, all mice recovered for at least 2 weeks before starting behavioral testing for maximal viral expression.

“Drinking in the Dark” (DID) procedures: In DID studies, a 4-day DID model was used, which is a standard protocol to induce binge-like ethanol consumption (Thiele and Navarro, 2014). This procedure involves replacing the cage water bottle with a 20% (v/v) ethanol solution or a 3% (w/v) sucrose solution 3-hours into the animals’ dark cycle. Drinking access each day was limited to 2 hours, with consumption recorded at the end of each access session. The amount consumed by each subject was recorded during hour 1 and hour 2 on the test (4th) day. Animals received a 3-day period of abstinence before the new 4-d cycle of DID began. For experiment 1, all animals went through two weeks of ethanol DID followed by two weeks of 3% (w/v) sucrose DID. For experiment 2, animals went through two cycles of ethanol DID followed by one cycle of sucrose DID. For experiment 3, 20 male and 20 female mice went through 6 consecutive cycles of ethanol DID, while the other 10 male and 10 female mice continued to consume normal drinking water. 10 male and 10 female DID ethanol mice were perfused directly after the final drinking session concluded, while the other 20 DID ethanol animals went through one 24-hour abstinence period before being sacrificed. On each perfusion day, half of the male and female water drinking control animals were also perfused.

2-bottle intermittent access to ethanol (IAE) procedures: For FIB-NPY animals, after completing three weeks of DID, the effect of NPY overexpression and secretion was assessed using the chronic intermittent access to ethanol (IAE) model, which has previously been shown to promote dependence-like phenotypes(Hwa et al., 2011). During IAE, animals receive two bottles on their home cage, with an alternating schedule of having two water bottles, and one water bottle one ethanol bottle. The ethanol solution used was 20% (v/v), and bottles were left on cages for 24 hours before being switched out. The ethanol bottle’s position was alternated daily to minimize place preference. Mice had access to ethanol for 24 hours on Monday, Wednesday, and Friday each week over a three-week period. To accurately calculate g/kg consumption, all animals were weighed on Mondays. Additionally, a dummy cage (without an animal) was placed on the same rack as the drinking animals to monitor potential bottle leakage during the 24-hour period. Any leakage recorded over a 24-hour period from either the ethanol or water bottle was then subtracted from animals’ drinking values on each respective bottle, to account for general fluid loss.

Open Field: Open field testing was used to determine if viral-mediated overexpression of NPY impacted animals’ locomotor or anxiety-like behaviors following DID and IAE testing. Animals were brought to the room with the locomotor chambers at least 30 minutes prior to testing and allowed to acclimate to the new environment. After the acclimation phase, animals were placed in the center of the testing chamber (42cm x 42 cm x 30cm; catalog #71-SFAC; Omnitech Electronics, Inc., Columbus, OH). All animals were left in the chambers for a 2-hour test period (the same length as access to ethanol during DID). Animals’ behavior and movements were recorded in 5-minute bins using VersaMax software (AccuScan Instruments, Inc., Columbus, OH) and quantified using VersaDat Version 4.00 (AccuScan Instruments, Inc.). For data analysis, differences in total distance traveled, as well as the time spent in the center of the test chamber during the first 5-minute bin were considered for differences between groups in locomotion and anxiety-like behavior.

Perfusion and Histology: For cannula placement checks, DREADD expression, and IHC, mice were prepared for perfusion by administering a 0.1mL/kg i.p. injection of ketamine/xylazine (6.67 mg/0.1 mL; 0.67 mg/0.1 mL; in 0.9% saline). Then, mice were perfused transcardially with 0.1M phosphate buffer saline (PBS; pH=7.4) and 4% paraformaldehyde. After extraction, brains were fixed in 4% paraformaldehyde for 24-48 hours, before being sliced at 40µm thickness on a vibratome (Leica VT1000S vibratome; Wetzlar, Germany). For the chemogenetic experiment, brain sections were mounted every other slice on glass charged slides (FisherBrand Superfrost, Thermo Fisher Scientific, Waltham, MA, USA) and allowed to air dry in the dark before being cover slipped using HardSet VECTASHIELD mounting medium (Vector Laboratories Inc., Burlingame, CA, USA). Then, cannula placement and mCherry-tagged DREADD expression were verified using a fluorescent microscope (Leica DM6000 B widefield light microscope, Leica Microsystems, Buffalo Grove, IL, USA). Cannula targets were defined by the end of the guide cannula plus 2mm to account for the injector tip. Only animals with bilateral mCherry expression and correct bilateral cannula placements were included for statistical analysis. 7 females and 9 males were included for the hM4d(Gi) group, and 12 females and 9 males met inclusion criteria for the mCherry control group. For the IHC experiment, at the time of slicing, random identification numbers were assigned to each brain to blind the experimenter to treatment conditions. Brain sections were stored in four sets at -20°C in cryopreserve solution until immunohistochemical staining.

Tissue Collection and Preparation (for experiment 2): Once FIB-NPY mice had concluded DID, IAE, and open-field testing, animals were euthanized via rapid decapitation for brain extraction and processing of brain tissue for RT-qPCR. Brains were extracted and flash frozen in O.C.T. compound (ThermoFisher Scientific) and were stored at -80°C until tissue slicing. A CM3050 S cryostat (Leica, Buffalo grove, USA) was used to slice brains at 40µm thickness until brain regions of interest were localized. Then, amygdalar brain punches were collected bilaterally for qPCR analysis via 1mm Integra™ Miltex® sterile disposable biopsy punches (VWR, Radnor, PA, USA). Each tissue punch was deposited in 2mL Precellys® CK14 bead tubes (Bertin Corp, Rockville, MD, USA) containing 500 µL of TriReagent (Molecular Research Center, Cincinnati, OH) and stored at -80°C until RNA extraction.

RNA extraction, cDNA synthesis, and RT-qPCR (for FIB-NPY experiment): The protocols for RNA extraction, cDNA synthesis, and RT-qPCR were based on methods described previously (Barkell et al., 2022). For RNA extraction, samples were thawed on ice, then homogenized (Precellys Instruments), and centrifuged to remove debris. The RNA-containing liquid was transferred to 2mL phase lock separation tubes (Cat# 2302830; QuantaBio), and 50 µL of BCP (1-Bromo-3-Chloropropane, Cat# BP 151; Molecular Research Center Inc.) was added. Samples were centrifuged for 15 minutes for phase separation, and the mRNA containing aqueous layer was transferred to new tubes. RNA purification occurred using the Qiagen RNEasy Tissue Mini Kit (Qiagen, Hilden, Germany). Each sample was washed with 70% ethanol, RW1 Buffer, and RPE Buffer respectively. After each wash, samples were centrifuged in butterfly tubes, and the wash that collected at the bottom of each tube discarded. The purified RNA was collected in 50 µL of RNAase free water, and samples were stored again at -80°C until RNA spectroscopy and cDNA synthesis.

RNA purity and concentration in each sample were assessed via RNA spectroscopy using the Take3 Application and Gen5 Software for Nucleic Acid Quantification (BioTek Instruments Inc.). Samples were loaded on the Take3 plate in duplicates, and RNA purity was indicated by an A260/280 value. Samples were re-run if not within a range of 10 ng/µL of each other. Before cDNA synthesis, each sample was diluted with PCR grade water depending on the concentration to achieve equal RNA concentrations. Then, cDNA was synthesized using the SuperScript IV VILO Master Mix (Invitrogen, ThermoFisher Scientific). 4 µL of Master Mix were added to each sample in a 96-well plate and heated using a Veriti 96 Well Fast Thermal Cycler (Applied Biosystems, ThermoFisher Scientific) at 25°C for 10 minutes, 50°C for 10 minutes, and 85°C for 5 minutes. Then cDNA samples were diluted with PCR grade water at a ratio of 1:2 and subsequently stored at -80°C until qPCR.

For qPCR, the TaqMan Fast Advanced Master Mix Kit (Applied Biosystems, ThermoFisher Scientific) was used. TaqMan Gene Expression Assays were used to assess mRNA levels for NPY, NPY receptors 1 and 2, and FIB-NPY. For the FIB-NPY assay, a custom TaqMan expression assay was built using ThermoFisher’s custom TaqMan assay design tool. Briefly, a sample of the FIB-NPY virus was sent to Plasmidsaurus (Eugene, OR) for viral sequencing. Then, the NPY peptide, NPY and FIB junction, and the FIB sequence (NCBI database; nucleotides 208–303, derived from the rat fibronectin mRNA sequence (Haberman et al., 2003) were identified. The target sequence spanning the full NPY and FIB sequences was entered into the TaqMan assay design tool (ThermoFisher Scientific), and the assay and primers were developed using ThermoFisher’s proprietary bioinformatics sequencing. BetaActin was used as the housekeeper gene and all mRNA expression levels for target genes were normalized to BetaActin mRNA expression. The Master Mix, Gene Expression Assay, and cDNA samples were pipetted into a 384-well plate for quantitative PCR (qPCR). The reaction proceeded in three phases. Initially, the samples were incubated at 50°C for two minutes to degrade potential PCR product contamination. This was followed by a 20-second incubation at 95°C to activate the polymerase. The amplification phase consisted of 45 cycles, each including denaturation at 95°C for one second and annealing/extension at 60°C for 20 seconds. Fluorescence data was collected at the end of each cycle using the QuantStudio Real-Time PCR Software.

Immunohistochemistry (for experiment 3): Four to six brain slices containing the CeA were collected from each animal and rinsed in 0.1M PBS (pH=7.4). Then tissue was blocked for 1 hour at room temperature in 3% normal donkey serum (Cat. No.: 0030-01; Southern Biotech, Birmingham, AL, USA), 0.3% Triton X100 (CAS No.: 9036-19-5; Sigma Aldrich, St. Louis, MO), and 0.1M PBS (pH=7.4), followed by 48-hour incubation at 5°C in primary rabbit anti-Y1R polyclonal antibody (1:500) (Ref No.: PA5-102698; Invitrogen, Rockford, IL) and primary goat anti-NeuN polyclonal antibody (1:500) (Ref. No.: PA5-143586; Invitrogen, Rockford, IL). Brain sections were again rinsed in 0.1M PBS (pH=7.4) three times for 5 minutes each. Then tissue was incubated in secondary antibody for 2 hours at room temperature in 3% normal donkey serum (Cat. No.: 0030-01; Southern Biotech, Birmingham, AL, USA), secondary donkey anti-rabbit antibody (Alexa Fluor™ 488; 1:1000) (Ref. No: A-21206; Invitrogen, Rockford, IL), secondary donkey anti-goat antibody (Alexa Fluor™ 647; 1:1000) (Ref. No: A-21447; Invitrogen, Rockford, IL), and 0.1M PBS (pH=7.4). After three more rinses in PBS, tissue was mounted on glass charged slides (FisherBrand Superfrost, Thermo Fisher Scientific, Waltham, MA, USA) and allowed to dry in the dark before being cover slipped using Fluoromount-G (Cat. No.: 0100-01; Southern Biotech, Birmingham, AL, USA). One tissue set was run through this IHC protocol without primary antibody to confirm lack of non-specific fluorescence.

Confocal Microscopy and Image Processing (for experiment 3): All images were acquired using a Zeiss LSM800 laser-scanning confocal microscope with a 2-track line of 488 nm and 640 nm diode laser lines (2 Gallium Arsenide Phosphide (GaAsP) detectors) and ZEN Blue software suite (Zeiss, Oberkochen, Germany). Z-stacks were acquired using a 63x oil-immersive objective, 1024 X 1024 frame size, 16-bit resolution, scan speed of 8, frame average of 2, and 1 µm step size. Six Z-stacks were taken per subregion, bilaterally per animal. The only adjustment for each image was the use of master gain to achieve the highest fidelity images per animal.

The following analysis method has been used previously to detect interleukin-1 colocalization with ionized calcium binding adaptor molecule 1 (IBA1) or an astrocytic maker in the dorsal hippocampus (Parekh et al., 2020). After imaging, AutoQuant X3 software (MediaCybernetics) was used to blindly deconvolve raw images; output files were exported to the Bitplane Imaris software suite (Zurich, Switzerland) for colocalization analysis. In Imaris, background correction was done for each channel individually using manually set absolute intensity thresholds. Each channel was then isolated, and a surface was built around it using the surface rendering function in Imaris software. A special masked channel was generated using these surfaces, which completely isolated the NeuN or NPY1R signal from background. In the colocalization module, voxels in both channels above the automatic threshold were included as colocalized voxels. A 2-D scatter plot was used to visually inspect the accuracy of colocalization thresholds and build a colocalization channel. Based on the colocalization analysis, the % of NPY1R signal volume (above background threshold) that overlapped with NeuN signal, representing the extent of Y1R expression within neurons was used for each Z-stack, and an average was calculated from the 6 bilateral Z-stacks per animal. Grubb’s outlier test was used to detect outliers within each animal’s set of images, as well as across animals of the same group. One animal was excluded from all analyses due to a bad IHC stain.

**Supplemental Results:**

Experiment 2.3: qPCR mRNA expression of Y1R and Y2R in the amygdala is not associated with individual differences in DID consumption.

For all graphics, dark colored data points are FIB-NPY animals (Supplemental Fig. 1A-B), and all light-colored data points are control animals (Supplemental Fig. 1C-D) (light/dark orange= female, light/dark blue=male). All data are collapsed across sex, unless there was a significant difference in linear regression when split by sex. For FIB-NPY animals there were no significant linear relationships between Y1R mRNA expression and average DID ethanol consumption week 1 (*F*(1, 32)=0.3772, *p*=0.5434; *R^2^*=0.0117), or week 2 (*F*(1, 32)=0.7214, *p*=0.402; *R^2^*=0.0221) (Supplemental Fig. 1A). Likewise, for FIB-NPY animals no significant linear relationships were seen for Y2R expression and ethanol intake week 1 (*F*(1, 32)=0.9949, *p*=0.326; *R^2^*=0.0302), week 2 (*F*(1, 32)=0.0015, *p*=0.9692; *R^2^*<0.0001) (Supplemental Fig. 1B). Control virus mice also did not indicate that Y1R mRNA expression could significantly predict ethanol DID drinking week 1 (*F*(1, 29)=0.6578, *p*=0.4239; *R^2^*=0.02218), week 2 (*F*(1, 29)=0.3184, *p*=0.5769; *R^2^*=0.0109) (Supplemental Fig. 1C). The same null results hold true for Y2R expression with ethanol DID week 1 (*F*(1, 29)=0.0263, *p*=0.8724; *R^2^*=0.0009), week 2 (*F*(1, 29)=0.08452, *p*=0.7733; *R^2^*=0.0029) (Supplemental Fig. 1D).

**Supplemental Figure:**


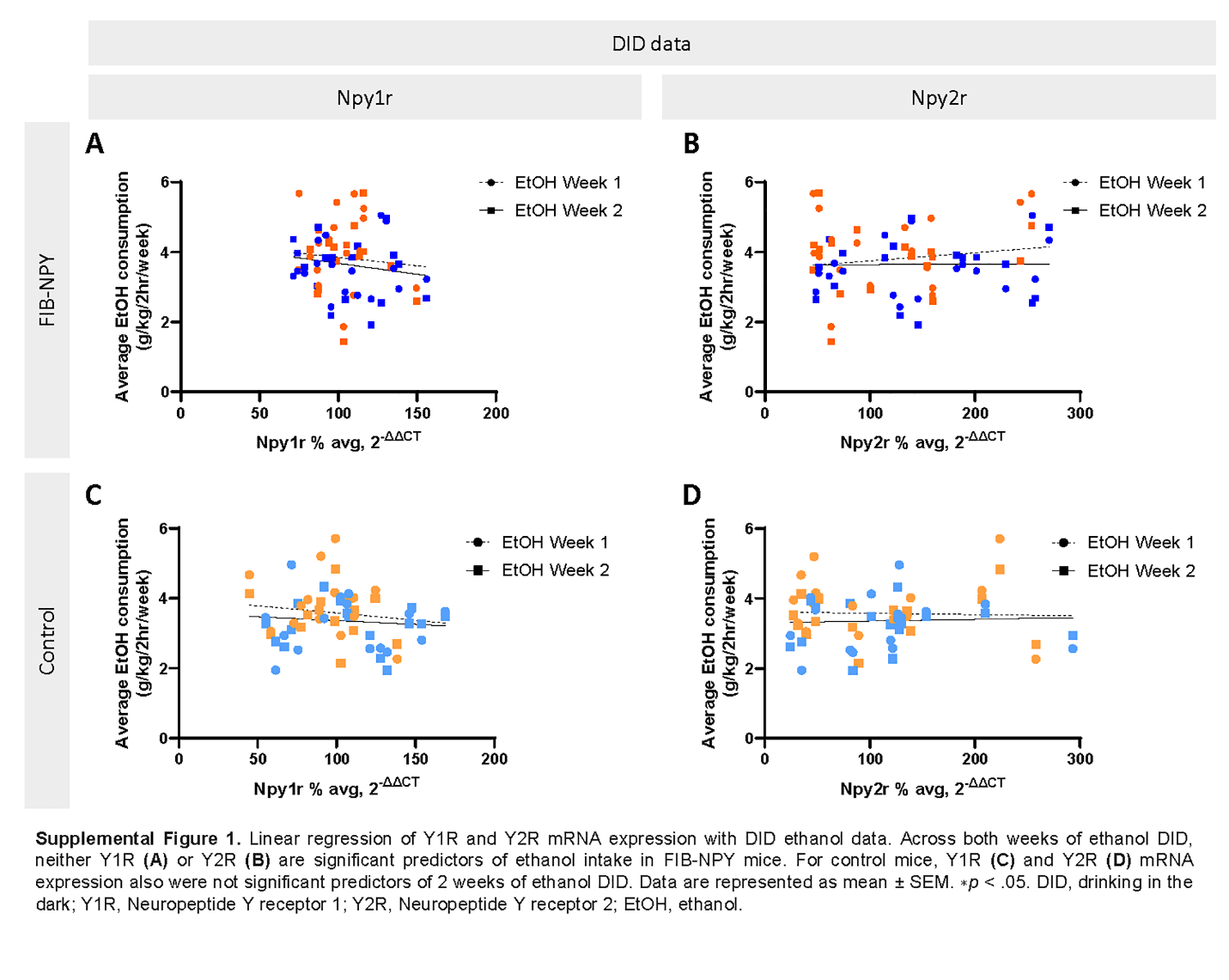


**References**

Barkell GA, Parekh SV, Paniccia JE, Martin AJ, Reissner KJ, Knapp DJ, Robinson SL, Thiele TE, Lysle DT (2022) Chronic ethanol consumption exacerbates future stress-enhanced fear learning, an effect mediated by dorsal hippocampal astrocytes. Alcohol Clin Exp Res 46**:**2177-2190.

Companion MA, Gonzalez DA, Robinson SL, Herman MA, Thiele TE (2022) Lateral habenula-projecting central amygdala circuits expressing GABA and NPY Y1 receptor modulate binge-like ethanol intake in mice. Addiction Neuroscience 3**:**100019-100019.

Haberman RP, Samulski RJ, McCown TJ (2003) Attenuation of seizures and neuronal death by adeno-associated virus vector galanin expression and secretion. Nature Medicine 2003 9:8 9**:**1076-1080.

Hwa LS, Chu A, Levinson SA, Kayyali TM, Debold JF, Miczek KA (2011) Persistent escalation of alcohol drinking in C57BL/6J mice with intermittent access to 20% ethanol. Alcoholism, clinical and experimental research 35**:**1938-1947.

McCown TJ (2006) Adeno-associated Virus-Mediated Expression and Constitutive Secretion of Galanin Suppresses Limbic Seizure Activity in Vivo. Molecular Therapy 14**:**63-68.

Parekh SV, Paniccia JE, Lebonville CL, Lysle DT (2020) Dorsal Hippocampal interleukin-1 signaling mediates heroin withdrawal-enhanced fear learning. Psychopharmacology 237**:**3653-3653.

Thiele TE, Navarro M (2014) "Drinking in the dark" (DID) procedures: a model of binge-like ethanol drinking in non-dependent mice. Alcohol 48**:**235-241.
